# Genomic Basis of Adaptation to a Novel Precipitation Regime

**DOI:** 10.1093/molbev/msad031

**Published:** 2023-02-15

**Authors:** Ahmed F Elfarargi, Elodie Gilbault, Nina Döring, Célia Neto, Andrea Fulgione, Andreas P M Weber, Olivier Loudet, Angela M Hancock

**Affiliations:** Molecular Basis for Adaptation Research Group, Max Planck Institute for Plant Breeding Research, Cologne, Germany; Université Paris-Saclay, INRAE, AgroParisTech, Institut Jean-Pierre Bourgin (IJPB), Versailles, France; Molecular Basis for Adaptation Research Group, Max Planck Institute for Plant Breeding Research, Cologne, Germany; Molecular Basis for Adaptation Research Group, Max Planck Institute for Plant Breeding Research, Cologne, Germany; Molecular Basis for Adaptation Research Group, Max Planck Institute for Plant Breeding Research, Cologne, Germany; Institute of Plant Biochemistry, Cluster of Excellence on Plant Science (CEPLAS), Heinrich Heine University, Düsseldorf, Germany; Université Paris-Saclay, INRAE, AgroParisTech, Institut Jean-Pierre Bourgin (IJPB), Versailles, France; Molecular Basis for Adaptation Research Group, Max Planck Institute for Plant Breeding Research, Cologne, Germany

**Keywords:** *Arabidopsis thaliana*, local adaptation, seasonal drought, stomatal conductance, water-use efficiency (WUE), mitogen-activated protein kinase 12 (MPK12)

## Abstract

Energy production and metabolism are intimately linked to ecological and environmental constraints across the tree of life. In plants, which depend on sunlight to produce energy, the link between primary metabolism and the environment is especially strong. By governing CO_2_ uptake for photosynthesis and transpiration, leaf pores, or stomata, couple energy metabolism to the environment and determine productivity and water-use efficiency (WUE). Although evolution is known to tune physiological traits to the local environment, we lack knowledge of the specific links between molecular and evolutionary mechanisms that shape this process in nature. Here, we investigate the evolution of stomatal conductance and WUE in an *Arabidopsis* population that colonized an island with a montane cloud scrubland ecosystem characterized by seasonal drought and fog-based precipitation. We find that stomatal conductance increases and WUE decreases in the colonizing population relative to its closest outgroup population from temperate North Africa. Genome-wide association mapping reveals a polygenic basis of trait variation, with a substantial contribution from a nonsynonymous single-nucleotide polymorphism in *MAP KINASE 12* (*MPK12* G53R), which explains 35% of the phenotypic variance in WUE in the island population. We reconstruct the spatially explicit evolutionary history of *MPK12* 53R on the island and find that this allele increased in frequency in the population due to positive selection as *Arabidopsis* expanded into the harsher regions of the island. Overall, these findings show how adaptation shaped quantitative eco-physiological traits in a new precipitation regime defined by low rainfall and high humidity.

## Introduction

Matching physiological traits to the environment is crucial for survival and reproductive success across diverse life forms. Under directional selection, distributions of traits in a population are expected to shift toward their new optima due to differential fitness over evolutionary time (Falconer and Mackay 1983; Lynch and Walsh 1998), resulting in the matching of a population's physiology to its environment (Stearns 1989; de Jong 1993; Zera and Harshman 2001; Roff 2002; Shefferson et al. 2003; Morrison and Stacy 2014; Villellas and García 2018). In animals, observations that metabolism, body size, and dimensions often vary with temperature are the basis of classic eco-physiological “rules” (Bergmann 1847; Allen 1877; Ray 1960; Dikmen et al. 2013; Lafuente et al. 2018; Zhou et al. 2018). In plants, photosynthesis is the major mode of energy acquisition, and the interface between the environment and constraints on photosynthesis is crucial. Here, form and function predict the economy of energy acquisition (Cowan 1986; Donovan and Ehleringer 1994), which in turn has been linked to spatial variation in selection pressures through associated physiological traits (Donovan and Ehleringer 1994; Wright et al. 2004; Díaz et al. 2016; Bjorkman et al. 2018). Overall, global distributions of traits involve optimization in the face of tradeoffs (Willi and Van Buskirk 2022).

In annual plants, flowering later can provide more time for the accumulation of resources, resulting in a potential fitness benefit (Korves et al. 2007). However, in ecosystems with seasonal drought, growing quickly to reproduce before the dry season may be favored (Cohen 1970; Ludlow 1989). But such rapid growth requires high levels of photosynthesis, which relies on gas exchange through stomata, the pores on the surface of leaves. For photosynthesis to occur, stomata must be open to allow gas exchange, reducing water-use efficiency (WUE) and making the plant vulnerable to drying (Geber and Dawson 1990, 1997). Leaf water loss through stomata is especially high in environments where the vapor pressure deficit, that is, the amount of air moisture relative to moisture-saturated air, is high (Will et al. 2013). Tuning the regulation of leaf pores, or stomata, is crucial for regulating the physiological tradeoff between increasing energy production via photosynthesis and water loss at the leaf surface (Moreno-Gutiérrez et al. 2012; Kenney et al. 2014; Querejeta et al. 2018). Therefore, in a given environment, optimal stomatal aperture in natural populations depends on the availability of moisture through rainfall as well as the vapor pressure deficit.

While precipitation is often considered to be synonymous with rainfall, in many regions, plants rely heavily on “horizontal” precipitation in the form of clouds or fog. These include the Lomas of Peru, fog deserts of Namibia, coastal western North American redwood forests and scrublands, and the seasonal montane cloud forests and scrublands of tropical Africa, Australia, and South America (Kerfoot 1968; Walter 1985; Stadtmüller 1987; Dawson 1998; Weathers 1999; Bruijnzeel et al. 2011; Karger et al. 2021). Such ecosystem types support a high proportion of Earth’s biodiversity, especially its endemic species (Bruijnzeel and Hamilton 2017). Understanding how plants adapt to these ecosystems is important for preserving biodiversity and identifying effective approaches to improve sustainable agriculture in these critical regions.

*Arabidopsis thaliana* is the major molecular model plant as well as an important eco-evolutionary model (Alonso-Blanco and Koornneef 2000; Koornneef et al. 2004; Mitchell-Olds and Schmitt 2006; Verslues and Juenger 2011; Weigel 2012; Assmann 2013). Eurasian populations of *A. thaliana* have been extensively studied and used to understand the genetic bases of adaptation to local environments (Fournier-Level et al. 2011; Hancock et al. 2011; Lasky et al. 2012; Exposito-Alonso et al. 2018; Ferrero-Serrano and Assmann 2019) and of variation in a wide range of traits related to development timing, metabolite and elemental content, pathogen response, growth and drought response (e.g., Atwell et al. 2010; Brachi et al. 2010; Chan et al. 2010; Li et al. 2010; Filiault and Maloof 2012; Davila Olivas et al. 2017; Kalladan et al. 2017; Zan and Carlborg 2019; Wieters et al. 2021; Bhaskara et al. 2022; Gloss et al. 2022; Roux and Frachon 2022). However, *A. thaliana* populations from North Africa (Brennan et al. 2014; Durvasula et al. 2017; Tabas-Madrid et al. 2018) and the Macaronesian archipelagos, including Madeira (Fulgione et al. 2018), the Canary Islands (Kranz and Kirchheim 1987), and the Cape Verde Islands (CVI; Fulgione et al. 2022; Tergemina et al. 2022), are mostly unstudied at the phenotypic level.

**Fig. 1.**
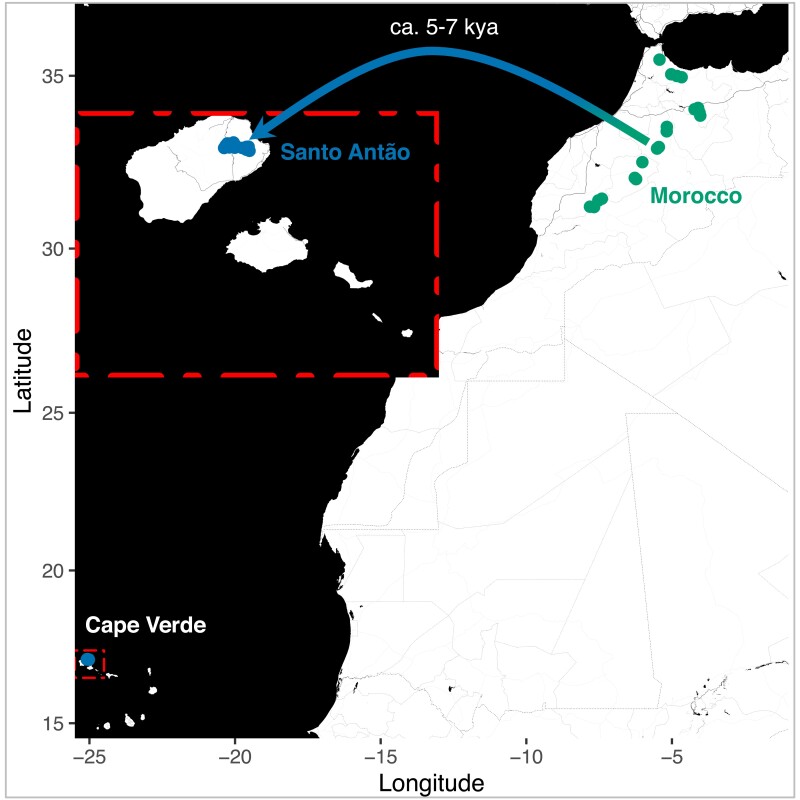
Collection locations of *Arabidopsis thaliana* Santo Antão, CVI (*n* = 189) and Morocco (*n* = 61). The arrow indicates the colonization of the CVI from Morocco ∼5–7 kya.

Here, we examine the evolution of stomatal conductance and WUE in a *A. thaliana* population that colonized the CVI. Islands can provide powerful systems for evolutionary analysis because they represent simplified “natural laboratories” where evolution can be studied in isolation (Losos and Ricklefs 2009). Such systems provided the basis for the theory of evolution by natural selection (Wallace 1855; Darwin 1859) and have been used to elucidate classic cases of adaptive processes (Losos et al. 1997; Grant 1999). *Arabidopsis thaliana* colonized CVI from temperate North Africa 5–7 kya through an extreme bottleneck that wiped out nearly all standing genetic variation (Fulgione et al. 2022; fig. 1). The CVI climate is defined by a short growing season with limited and highly variable rainfall. *Arabidopsis thaliana* in Cape Verde is restricted to high altitude (>950 m) north-facing slopes, where vegetation is bathed in moisture derived from humid trade winds (Brochmann et al. 1997; Fulgione et al. 2022). The short growing season combined with high humidity creates an environment that differs substantially from the Mediterranean climate of the Moroccan Atlas Mountains, which supports the closest outgroup populations (fig. 4; supplementary fig. S2, Supplementary Material online in Fulgione et al. 2022).

In this study, we find a shift in the phenotype distribution toward higher stomatal conductance and lower WUE in Cape Verde relative to North Africa. Using genome-wide association mapping, we characterize the trait architecture and identify a nonsynonymous variant (G53R) in the *MPK12* gene that explains a large proportion of the trait variation. We then reconstruct the historical spread of this variant across the island and find evidence that the derived allele facilitated local adaptation to the new tropical precipitation regime defined by limited rainfall and moisture delivered primarily through high air humidity.

## Results

### Stomatal Conductance is Higher and Water Use Efficiency Lower in CVI Compared with Morocco

In CVI, rainfall is limited and unpredictable (supplementary fig. S1*A* and *B*, Supplementary Material online), and water vapor pressure (specific humidity) is consistently high relative to Moroccan *A. thaliana* sites (supplementary fig. S1*C*, Supplementary Material online). The median relative humidity across CVI sites during the growing season is 86.9% with lower and upper bounds of 65.5–95.9% (supplementary fig. S2, Supplementary Material online).

We hypothesized that local adaptation may have acted to optimize performance in CVI *Arabidopsis* populations in response to the shift to higher humidity here. To investigate this possibility, we examined variation in WUE (measured as carbon isotope discrimination, δ^13^C) and stomatal conductance (gas-exchange capacity) in “well-watered” (WW) and moderate water-deficit (WD) conditions in 152 lines from the CVI of Santo Antão and 24 representative Moroccan outgroup lines (supplementary table S1, Supplementary Material online). In large-scale phenotyping experiments, it is challenging to consistently apply drought stress conditions across pots because of spatial heterogeneity in drying rates. To deal with this, we used the high throughput Phenoscope platform that automatically circulates pots and adjusts watering several times per day based on pot weight, allowing experiments that would not be practical with manual procedures (Tisné et al. 2013).

**Fig. 2.**
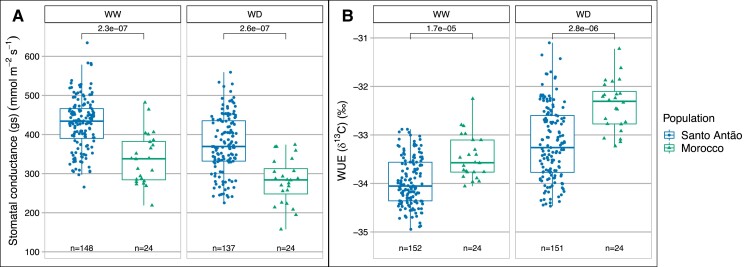
Phenotypic variation in (*A*) stomatal conductance (gs) and (*B*) WUE for Santo Antão, CVI, and Moroccan *Arabidopsis thaliana* populations in WW and WD conditions. The line in the center of the boxplots represents the median, the box edges represent the 25th and 75th percentiles (lower and upper bound, respectively), and the whiskers represent the 95% CI. The WUE is measured as carbon isotope discrimination (δ^13^*C*), and the carbon isotope ratio is expressed per mil, ‰. The *P*-values for the Mann–Whitney–Wilcoxon test are shown.

We examined the effects of drought treatment and geographic origin on stomatal conductance and WUE. The WD condition led to an average of 40% less rosette growth at the end of the experiment compared with WW, indicating that the WD condition reduced growth rate on average. Average stomatal conductance was higher in the Santo Antão (CVI) population than in the Moroccan population in both watering conditions (WW: LMM, region fixed-effect estimate = 88.16 mmol/m^2^ s, *P* < 0.001; WD: LMM, treatment fixed-effect estimate =−53.6 mmol/m^2^ s, *P* = 0.011; supplementary table S2, Supplementary Material online; fig. 2*A*), and WUE was reduced in the Santo Antão population relative to the Moroccan outgroup population in both conditions (WW: LMM, region fixed-effect estimate = −0.44‰, *P* = 0.003; WD: LMM, treatment fixed-effect estimate = 1.6‰, *P* < 0.001; fig. 2*B*). As expected, WUE was strongly negatively correlated with stomatal conductance across Santo Antão lines (Pearson correlation coefficient *R*^2^ = 0.23, *P* = 8.3 × 10^−10^, and *R*^2^ = 0.28, *P* = 5.5 × 10^−11^, for the WW and WD treatments, respectively; supplementary fig. S3, Supplementary Material online). Overall, trait distributions shifted such that in the seasonally humid Santo Antão population, mean stomatal conductance was higher and mean WUE lower than in the Moroccan population. The shifts in the distributions were similar across populations, resulting in parallel reaction norms with consistent genetic differences in both treatments, which imply a simple genetic response, with no evidence of a genotype by environment (GxE) interaction (supplementary fig. S4, Supplementary Material online).

### WUE and Stomatal Conductance are Moderately Polygenic

The high trait variation we observed within the Santo Antão population suggested that the genetic variation responsible for these traits may segregate there. The proportion of trait variance attributable to genetic variation, or heritability, provides information about the potential for genetic mapping within a natural population. We estimated heritability based on the proportion of the phenotypic variance explained by all genotyped single-nucleotide polymorphisms (SNPs), which is commonly referred to as “chip heritability” (Zhou and Stephens 2012; Zhou 2014). The estimated heritability was moderate for stomatal conductance (0.45, 95% confidence interval [CI] 0.29–0.60 for average stomatal conductance across conditions, 0.40, 95% CI 0.22–0.59 in WW, and 0.29, 95% CI 0.12–0.46 in WD) and high for WUE (0.82, 95% CI 0.75–0.88 for the average WUE, 0.30, 95% CI 0.14–0.47 for the drought response [the difference between WD and WW conditions], 0.81, 95% CI 0.73–0.87 in WW, and 0.73, 95% CI 0.63–0.81 in WD). This discrepancy may imply that WUE is impacted less by uncontrolled environmental variation than stomatal conductance or that the genetic basis of stomatal conductance variation is more complex and not captured as well by additive genetic variance models. Moreover, stomatal conductance is an instantaneous measure, and WUE measured as the carbon isotope ratio is an integrated measure over the lifetime of the leaf, and thus may be expected to have higher heritability.

**Fig. 3.**
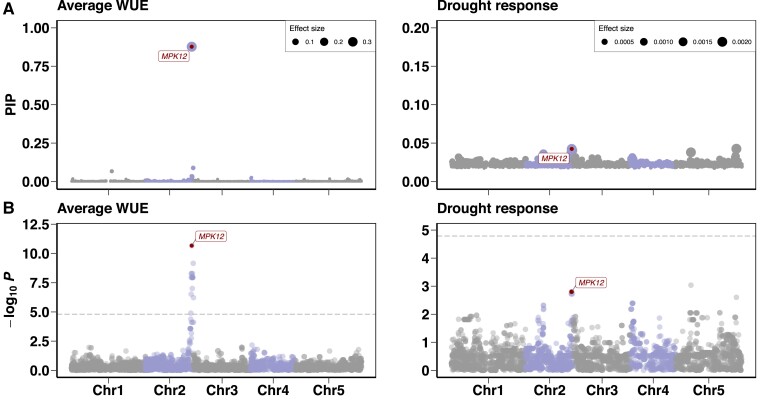
Genome-wide association (GWA) mapping of WUE. (*A*) Polygenic modeling of the average WUE across the WW and WD conditions (left) and the drought response of WUE (the difference between both conditions: WW-WD) (right) in the Santo Antão *Arabidopsis thaliana* population using BSLMM. The *y*-axis represents the posterior inclusion probability and the size of symbol denotes the effect size. (*B*) Genome-wide association mapping of average WUE (left) and its drought response (right) using LMM. The horizontal dashed line corresponds to the Bonferroni significance threshold at *α* = 0.05. In (*A*) and (*B*), points represent SNPs along the five chromosomes. The red point at chromosome 2 represents the *MPK12* G53*R* variant (a substitution of arginine for glycine at amino acid position 53).

Next, we investigated the genetic architecture of the traits using a Bayesian sparse linear-mixed model (LMM) that allows for a mixture of large and infinitesimal genetic effects (Zhou et al. 2013). We found that seven loci explained 82% (95% CI 75–88%) of the genetic variance for average WUE and 68 loci explained 30% (95% CI 14–47%) of the genetic variance for the drought response of WUE (fig. 3*A*, supplementary tables S3 and S4, Supplementary Material online). Furthermore, we found that seven loci were predicted to have effects on WUE in WW and WD conditions (supplementary fig. S5*A*, tables S3 and S4, Supplementary Material online). For the genetic architecture of the average stomatal conductance and the drought response, we found about that 39 and 44 loci have a major effect, respectively (supplementary fig. S6*A*, tables S3 and S4, Supplementary Material online). In addition, about 39 and 53 loci were predicted to have major effects in WW and WD conditions, respectively (supplementary fig. S7*A*, tables S3 and S4, Supplementary Material online). We also examined the strength of genetic correlation between WUE and stomatal conductance, which reflects the average effect of pleiotropic action across all causal loci in both traits and helps to describe their complex relationships (van Rheenen et al. 2019). We observed a negative genetic correlation (Pearson correlation coefficient *R*^2^ = 0.12, *P* < 2.2 × 10^−16^ for the average traits, *R*^2^ = 0.28, *P* < 2.2 × 10^−16^ for the drought response of traits, *R*^2^ = 0.16, *P* < 2.2 × 10^−16^ for WW, and *R*^2^ = 0.55, *P* < 2.2 × 10^−16^ for WD) between both traits across Santo Antão lines. Overall, we found that the genetic architecture was moderately complex for WUE and stomatal conductance and that a significant fraction of the genetic basis for the traits is shared between traits based on their genetic correlations.

### A Nonsynonymous Variant in *MPK12* (G53R) Explains a Large Proportion of Trait Variance

To identify specific loci underlying variation in the average traits, drought response of traits, and both conditions, we used a LMM approach that controls for population structure by including a relatedness matrix in the model (Zhou and Stephens 2014). For the average WUE based on δ^13^C measurements, we detected a single Bonferroni significant peak on the end of chromosome 2 (fig. 3*B*) as well as in WW and WD conditions (supplementary fig. S5*B*, Supplementary Material online). This peak contains a nonsynonymous variant (G53R) in the *Arabidopsis mitogen-activated protein kinase12* (*MPK12*; AT2G46070) gene, which was previously implicated in WUE in Cvi-0×L*er*-0 RIL and NIL mapping populations (Juenger et al. 2005; Des Marais et al. 2014). Further, we found that one of the highest peaks contained the *MPK12* region in the drought response of WUE (fig. 3*B*). This variant explained 35% of the variation in the average WUE, 10% for drought response, 33% for WW, and 29% for WD in the Santo Antão population.

We identified several potentially interesting associations in addition to *MPK12* across the genome in the drought response of WUE. One of the highest peaks on chromosome 5 (drought response of WUE; fig. 3*B*) contains *PBL27* (AT5G18610), which encodes a receptor-like cytoplasmic kinase that is required to phosphorylate the SLOW ANION CHANNEL-ASSOCIATED HOMOLOG 3 (SLAH3) for antifungal immunity and chitin-induced stomatal closure. It has been shown that this signal transduction is independent of ABA-induced SLAH3 activation (Liu et al. 2019). Another genomic region on chromosome 5 comprises a downstream gene variant mapped to the *CNX1* gene. CNX1 catalyzes the final step of the synthesis of molybdenum cofactor (MoCo), a cofactor for multiple plant enzymes: abscisic acid (ABA), auxin, and nitrate (Porch et al. 2006). Another peak on chromosome 1 contained an upstream gene variant in *WRKY57*, a gene for which increased expression was previously shown to improve drought tolerance in *Arabidopsis* through increased ABA (Jiang et al. 2012). A peak at the top of chromosome 4 contained the well-known *FRIGIDA* (*FRI*) K232X variant, a major determinant of flowering time in *A. thaliana* (Johanson et al. 2000; Gazzani et al. 2003; Shindo et al. 2005; Fulgione et al. 2022). Lovell et al. (2013) showed that the derived *FRI* allele pleiotropically confers a drought-escape strategy through decreased flowering time, decreased WUE, and increased growth rate. We also identified an association peak corresponding with a stop-loss variant in the *ER-type Ca2+-ATPase 2* (*ECA2*) gene, which catalyzes the efflux of calcium from the cytoplasm. The cuticle mutant *eca2* that has an altered phenotype in cutin and wax showed a plant defense response to different biotic stresses, including biotrophic and necrotrophic pathogens and herbivory insects (Blanc et al. 2018; Aragón et al. 2021). Since some of these associations could arise due to partial linkage disequilibrium with the major effect variant at *MPK12*, we calculated the proportion of variance explained with and without *MPK12* G53R as a covariate. The PVE was reduced for *PBL27* (9–8%), *CNX1* (8–2%), and *WRKY57* (4–2%), unchanged for *ECA2* (6%), and the PVE increased for *FRI* (4–6%) with *MPK12* G53R as a covariate. Overall, these results support a moderately polygenic architecture for the drought response of WUE.

For stomatal conductance, GWAS revealed no Bonferroni significant results; however, the highest peaks in the average stomatal conductance (supplementary fig. S6*B*, Supplementary Material online) as well as for both conditions separately (supplementary fig. S7*B*, Supplementary Material online) contained the *MPK12* region. Here, the proportion of the genetic variance explained by *MPK12* G53R was 10% in the average stomatal conductance, 7% in the WW condition, and 12% in the WD condition. Plants from the natural population carrying the derived *MPK12* 53R allele had lower WUE and higher stomatal conductance than those carrying the ancestral G53 allele (supplementary fig. S8*A* and *B*, Supplementary Material online). Taken together, our results support a central role for the *MPK12* G53R variant in trait variation in the natural CVI population.

### Reconstructing the Evolutionary History of Variation in WUE

#### Population Structure in Santo Antão

**Fig. 4.**
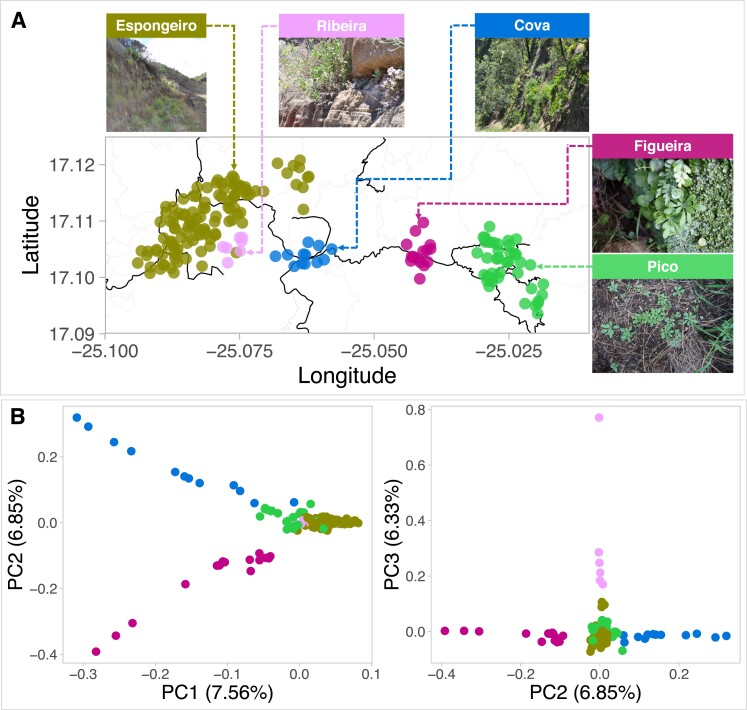
Population structure of *Arabidopsis thaliana* subpopulations in Santo Antão. (*A*) Geographical distribution of subpopulations across Santo Antão. Images of representative sites for the five subpopulations in Santo Antão show the diversity of habitats that *A. thaliana* occupies. (*B*) PCA of genome-wide SNPs showing clustering within Santo Antão. Abbreviations: Figueira: Lombo de Figueira; Cova: Cova de Paúl; Ribeira: Ribeira de Poio; Pico: Pico da Cruz.

As a first step toward reconstructing the evolutionary history of WUE variation in Santo Antão, we examined the overall population structure of *A. thaliana* on the island. We found that the Santo Antão population could be divided into five major subpopulations based on results from principal component analysis (PCA) and neighbor-joining tree using LD-pruned genome-wide SNP variation (fig. 4*A* and *B*, supplementary fig. S9*A*, Supplementary Material online). The subpopulations include Lombo de Figueira, Cova de Paúl, Ribeira de Poio, Pico da Cruz, and Espongeiro, which are hereafter referred to as Figueira, Cova, Ribeira, Pico, and Espongeiro. *Arabidopsis thaliana* plants in Santo Antão tend to be found on rock outcrops and to be restricted to Northeast-facing slopes, where they are exposed to humid northeasterly trade winds. This produces an east-west cline such that precipitation is highest and the growing season longest on the north-eastern side of the island, at the sites Figueira, Cova and Pico, and the growing season is shorter in the more western Ribeira and Espongeiro sites (Brochmann et al. 1997).

In the PCA, the Cova and Figueira subpopulations split on the first principal component axis, consistent with the previous finding that they represent the most ancestral variation in Santo Antão (Fulgione et al. 2022). Although the Ribeira subpopulation lies geographically near the Espongeiro subpopulation, it splits from Espongeiro on the second PC (fig. 4*B*). The third PC further distinguishes lines within Espongeiro. Conversely, the two geographically separated subpopulations, Pico and Espongeiro, appear to be closely related despite their large geographic distance, suggesting recent spread and ongoing migration. These results are consistent with subpopulation split times inferred previously (supplementary fig. S8, Supplementary Material online in Fulgione et al. 2022), and with results from subpopulation topologies we inferred across 50-SNP genomic windows with *Twisst* (Martin and Van Belleghem 2017). The results showed that the most common topology across the genome grouped Espongeiro and Pico, followed by Ribeira and then Figueira (Cova, Figueira, Ribeira [Espongeiro, Pico]; supplementary fig. S9*B*, Supplementary Material online). Overall, these results support a deep split between Cova and Figueira and a more recent expansion into the disjunct Espongeiro and Pico, with continuing gene flow between these regions.

#### Evolutionary History of Genetic Variation in Water Use Efficiency

**Fig. 5.**
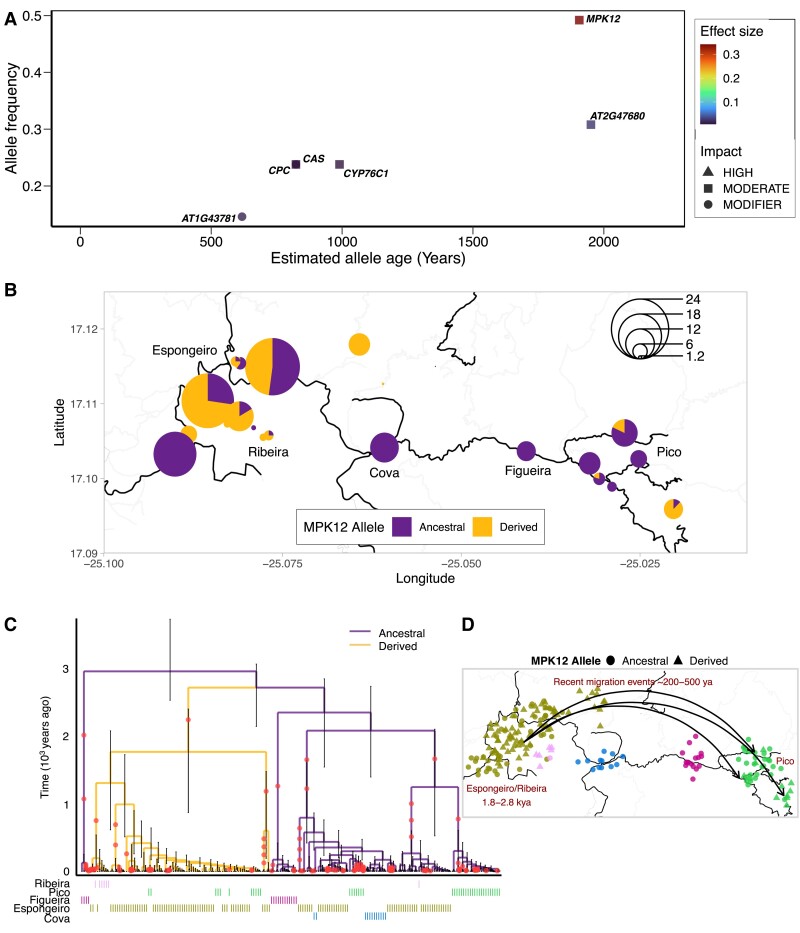
Evolutionary history of water-use efficiency variation. (*A*) Estimated allele ages (inferred in RELATE) versus allele frequencies of variants with major effects estimated from GWA mapping of average WUE. Shape denotes predicted impact from gene annotation. (*B*) Spatial distribution of *MPK12* G53*R* in Santo Antão. Pie charts show the frequency of *MPK12* alleles, with size representing the number of individuals per sampling location. (*C*) Marginal genealogical tree estimated in RELATE for *MPK12* G53*R*. (*D*) A model of the origin and spread of the *MPK12* G53*R* variant based on the genealogical inference in (*C*). Figueira: Lombo de Figueira; Cova: Cova de Paúl; Ribeira: Ribeira de Poio; Pico: Pico da Cruz.

We next investigated the evolutionary history of the WUE trait in the Santo Antão natural population. We estimated the ages of loci associated with average WUE and found that the derived *MPK12* 53R allele was one of the first to arise. We estimated the age of *MPK12* 53R to be between 1.8 kya (time to the allele's most recent common ancestor; 95% CI 0.87–2.4 kya) and 2.8 kya (based on allelic divergence; 95% CI 2.2–3.1 kya; fig. 5*A*). Overall, our results are consistent with a model where the strong effect *MPK12* 53R variant arose early relative to other variants that impact WUE.

The *MPK12* G53R variant segregates at intermediate frequency (43%) in Santo Antão and exhibits structure across subpopulations (fig. 5*A* and *B*, supplementary fig. S10, Supplementary Material online). *MPK12* 53R is absent in the Figueira and Cova subpopulations, which represent the initial extent of the *A. thaliana* distribution in Santo Antão before expansion into the drier Espongeiro region at ∼3 kya (supplementary fig. S8, Supplementary Material online in Fulgione et al. 2022). The complete absence of *MPK12* 53R in the early splitting Cova and Figueira, together with the age estimate for *MPK12* 53R, suggests that the allele likely arose after the split from these subpopulations. Among the more recently expanded subpopulations, the *MPK12* 53R allele varies in frequency across sites along an east-to-west gradient. The frequency of the derived allele is highest in the western-most subpopulations (Ribeira [90%] and Espongeiro [53%]) and lower in the moister eastern Pico region (29%).

**Fig. 6.**
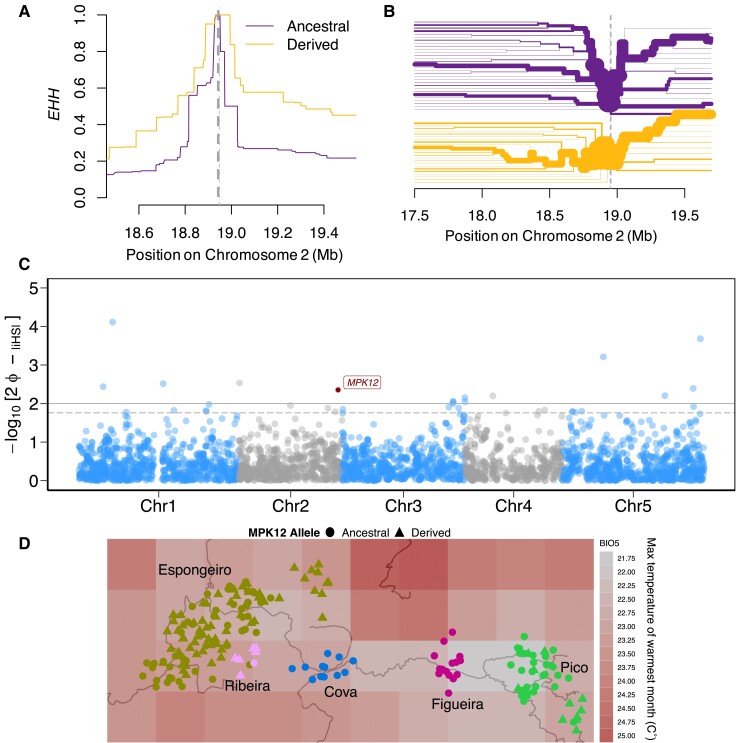
Signature of a partial selective sweep at *MPK12* G53*R*. (*A*) The decay of EHH and (*B*) bifurcation analysis of the ancestral (upper) and derived (lower) alleles for *MPK12* G53*R*. The dotted line marks the position of the focal *MPK12* SNP. The width of the lines in (*B*) represents the frequency of haplotypes bearing the ancestral and derived *MPK12* variant. (*C*) Genome-wide integrated haplotype score (*iHS*) analysis for the Santo Antão population. Horizontal solid line represents the significance threshold applied to detect the outlier SNPs (−log_10_ (*P*) = 2) and the horizontal dashed line represents the 1% tail based on the genome-wide empirical distribution. The *MPK12* 53*R* variant is marked with a dark point and a box with the text “*MPK12*”. (*D*) Geographic distribution of Santo Antão *Arabidopsis thaliana* individuals overlaid on the max temperature of warmest month (BIO5). Abbreviations: Figueira: Lombo de Figueira; Cova: Cova de Paúl; Ribeira: Ribeira de Poio; Pico: Pico da Cruz.

To better understand the origin and historical spread of the *MPK12* 53R variant across the island, we examined the genealogical relationships between populations and individuals for the genomic region linked to this variant. The maximum likelihood topology for the 50-SNP window centered on the *MPK12* locus matched the major genome-wide topology (Cova, Figueira, Ribeira [Espongeiro, Pico]; supplementary figs. S9*B* and S11, Supplementary Material online). To examine the relationships at the scale of individual lines, we produced a marginal genealogical tree for the region using RELATE v1.1.4 (Speidel et al. 2019; fig. 5*C*). The deepest branches of the derived *MPK12* 53R haplotype are found in the Ribeira and Espongeiro subpopulations, suggesting this allele first arose and rose to high frequency there. Clustering of individuals within the genealogical tree and the frequency distribution across the island suggest that *MPK12* 53R spread through multiple migrants into the Pico subpopulation in the past few hundred years (fig. 6*D*). However, the *MPK12* 53R allele frequency has remained low in the moister Pico region. The allele frequency difference across populations suggested that *MPK12* 53R may be favored in the warmer, more exposed Ribeira/Espongeiro region, where rapid growth to escape drought would be most important.

#### Evidence for Adaptive Evolution at *MPK12* G53R

We next asked whether there was evidence the *MPK12* 53R allele was adaptive in Santo Antão. When an allele is driven quickly to high frequency in a population due to a partial selective sweep, a signature of an extended haplotype with reduced linked variation is expected (Hudson et al. 1994). Consistent with this, we identified an extended region of high haplotype homozygosity (EHH; Sabeti et al. 2002) for the core-derived *MPK12* 53R allele relative to the ancestral *MPK12* G53 allele (fig. 6*A* and *B*). To determine whether this locus is an outlier for haplotype homozygosity relative to the genome as a whole, we calculated the integrated haplotype score (*iHS*; Voight et al. 2006) across the genomes of the Santo Antão population. We found that *iHS* for the *MPK12* locus is extreme compared with the genome-wide distribution of haplotype homozygosity (|*iHS*| = 2.85, −log_10_[*P*-value] = 2.35; fig. 6*C*).

We next used gene ontology (GO) enrichment to assess evidence of selection on traits based on the *iHS* results. Since the overall genetic variation in Santo Antão is low, the number of genes with *iHS* signals is also limited. Therefore, we did not expect to have high power in a GO enrichment analysis. Still, we found a marginally significant enrichment for several biological processes. GO analysis revealed enrichment in genes regulating stomatal closure, abscission, osmotic stress, salicylic acid–mediated signaling, transcription elongation from RNA polymerase II promoter, regulation of DNA-templated transcription elongation, transition to flowering, auxin-activated signaling pathway, cellular response to an organic substance, and signal transduction (supplementary fig. S12*A*, *B* and table S5, Supplementary Material online). Enrichments in stomatal closure, osmotic stress, salicylic acid signaling, response to organic substances, and signal transduction were largely driven by the same set of three genes. These included *MPK12*, the defense-related transcription factor *WRKY54*, and *AUXIN RESPONSE FACTOR 2* (*ARF2*). Enrichment of the transition to the flowering category was also driven by three genes: *CLAVATA 2* (*CLV2*), *EMBRYONIC FLOWER 1* (*EMF1*), and *ARF2*. Overall, these results suggest that both flowering time and water balance may have been important selection pressures in the CVI population.

Further, a test of cross-population EHH (XP-EHH; Sabeti et al. 2007) showed the derived haplotype in Espongeiro is highly differentiated with elevated haplotype homozygosity compared with the ancestral Cova subpopulation (|XP-EHH| = 2.16, −log_10_[*P*-value] = 1.52; supplementary fig. S13*A*, Supplementary Material online) as well as between Espongeiro and Pico (|XP-EHH| = 2.03, −log_10_[*P*-value] = 1.37; supplementary fig. S13*B*, Supplementary Material online). We estimated a selection coefficient of 4% for the variant based on the inferred allele frequency trajectory from the local inferred genealogy (fig. 5*C*, supplementary fig. S14 and table S6, Supplementary Material online). To control for population growth during this timeframe, we estimated the selection coefficient for *MPK12* 53R against the previously inferred trajectory of historical population size (*N_e_*) using whole-genome trees (Fulgione et al. 2022). Overall, these findings are consistent with positive selection acting on the derived *MPK12* allele in populations that expanded into the harsher western Ribeira/Espongeiro region of the island.

Environmental correlation analysis can provide further evidence for local adaptation based on statistical associations between climate variables and genetic variants (Hancock et al. 2011; Lasky et al. 2012). To determine whether local adaptation to climate might have shaped the frequency of *MPK12* 53R allelic variation across populations, we conducted a partial redundancy analysis (RDA). RDA links genomic variation to environmental predictors while accounting for geographic population structure by including geographic distance as a model covariate. We found a significant association between climate and genomic variation overall (*P* = 0.001; *R*^2^ = 0.34; adjusted *R*^2^ = 0.313) and applied a stepwise model-building algorithm (*ordistep*) to determine which bioclimatic variables (supplementary table S7, Supplementary Material online) best explained the spatial distribution of the genetic data. Five environmental variables (BIO5: Max Temperature of Warmest Month, BIO11: Mean Temperature of Coldest Quarter, BIO13: Precipitation of Wettest Month, BIO17: Precipitation of Driest Quarter, BIO19: Precipitation of Coldest Quarter) explained a large proportion of the variance across populations (*P* = 0.001; *R*^2^ = 0.15; adjusted *R*^2^ = 0.127; supplementary fig. S15, tables S8 and S9, Supplementary Material online). Espongeiro and Ribeira subpopulations separated from Figueira, Cova, and Pico on the first RDA axis, which was associated with the temperature variables (BIO5 and BIO11; supplementary fig. S15*A*, Supplementary Material online), whereas variation that separated subpopulations Figueira, Cova, and Pico loaded on the second RDA and was associated with the precipitation variables (BIO13, BIO17, and BIO19; supplementary fig. S15*A*, Supplementary Material online). We next examined the loadings by SNP (supplementary fig. S15*B* and table S10, Supplementary Material online) to determine whether the *MPK12* 53R SNP variant was correlated with the partial RDA loadings. We found that *MPK12* G53R was an outlier in the RDA1 SNP loadings, and its distribution was most strongly predicted by BIO5, the maximum temperature of the warmest month (fig. 6*D*; supplementary fig. S15*C*, Supplementary Material online). This suggests that the *MPK12* 53R variant is adaptive in the warmest microclimates in Santo Antão, in Espongeiro, and Ribeira, where the growing seasons are shortest and the need for increased photosynthesis and faster growth may be strongest.

Finally, we asked whether the population genetic evidence we found for positive selection translated to a reproductive advantage in an experimental setting. To determine whether *MPK12* G53R was associated with differential fitness in a simulated Santo Antão environment, we used fitness data (total number of seeds produced) from plants we propagated in a growth chamber set to simulate humidity, air and soil temperature, soil chemistry and precipitation, photoperiod, and light availability of an Espongeiro site in Santo Antão (Fulgione et al. 2022). We observed that plants carrying the derived *MPK12* 53R variant produced more seeds than plants with the ancestral *MPK12* G53 variant (negative binomial generalized linear model [GLM], *MPK12* 53R allele fixed-effect estimate = 0.76, *P* = 0.00332; supplementary fig. S16 and table S11, Supplementary Material online). Since we previously found that flowering time was strongly associated with fitness in the CVI-simulated environment (Fulgione et al. 2022) and because flowering time and WUE have been implicated in drought avoidance (Mooney et al. 1976; Geber and Dawson 1990, 1997; Donovan and Ehleringer 1992; McKay et al. 2003; Heschel and Riginos 2005; Sherrard and Maherali 2006), we also examined the effect of *MPK12* G53R while controlling for *FRI* K232X. In a GLM with a negative binomial transformation of seed number, the signal for *MPK12* G53R on fitness was reduced but still highly significant (GLM, *MPK12* 53R allele fixed-effect estimate = 0.7, *P* = 0.00519) (supplementary table S12, Supplementary Material online), indicating the *MPK12* 53R variant increases fitness independently from *FRI* 232X under CVI (Espongeiro) conditions.

## Discussion

We examined the evolution of stomatal conductance and WUE in an *A. thaliana* population that colonized a novel precipitation regime. We found that average stomatal conductance increased and WUE decreased in the humid CVI population relative to the North African outgroup. We found that trait architecture was polygenic, with an important contribution from a nonsynonymous variant (G53R) in *mitogen-activated protein kinase 12* (*MPK12*), which explained 35% of the trait variance in WUE in the Santo Antão island population. We found evidence that the derived *MPK12* 53R variant is evolving under positive selection based on its association with temperature across the island (fig. 6*D*; supplementary fig. S15*B* and *C*, Supplementary Material online) and on a haplotype-based signature of selection in the genomic region (fig. 6*A*–*C*). Finally, we found that the derived *MPK12* 53R variant conferred higher fitness than the ancestral *MPK12* G53 variant in plants grown in CVI conditions (supplementary fig. S16, Supplementary Material online). Overall, our findings reveal evidence that the *MPK12* 53R variant helped facilitate local adaptation on the island of Santo Antão, where “horizontal” precipitation, or fog, is an important contributor to total precipitation.

Our findings are also relevant in the context of understanding how plants adapt to seasonal drought and the importance of physiological tradeoffs more generally. Plants use different strategies to maintain water balance (Klein 2014; Martínez-Vilalta et al. 2014; Skelton et al. 2015). Most plants are isohydric; they avoid reaching low water potential by closing their stomata during drought. However, in environments where humidity is reliably high and the vapor pressure deficit is low, plants may be anisohydric, keeping their stomata open even when rainfall is limited. Rainfall in CVI is unpredictable, but trade winds provide a steady supply of high humidity to plants growing along the northeast-facing slopes during the short growing season (supplementary figs. S1 and S2, Supplementary Material online). In the humid regions of the island of Santo Antão in Cape Verde, where *A. thaliana* is found, the anisohydric strategy may be common. Our results indicate that *A. thaliana* populations here have evolved an anisohydric strategy in response to the humid environment.

This anisohydric strategy may provide other benefits. In drought-prone environments, plant populations may adapt by escaping drought (Levitt 1972; Ludlow 1989). When growing seasons are short, plant populations may maximize fitness by increasing stomatal conductance to increase rates of carbon gain through photosynthetic carbon assimilation and thus escape drought stress (Mooney et al. 1976; Geber and Dawson 1990, 1997; Donovan and Ehleringer 1992; McKay et al. 2003; Heschel and Riginos 2005; Sherrard and Maherali 2006). A drought-escape strategy appears to be strongly favored by selection in the CVI (Fulgione et al. 2022), where growing seasons are short. More open stomata may enable higher levels of photosynthesis and faster growth, facilitating such a drought-escape strategy. In this case, the high relative humidity may effectively reduce the tradeoff between photosynthesis and transpiration. Overall, our findings reveal a case where natural selection appears to have optimized carbon gain through increased stomatal aperture, facilitating drought escape in a natural population.

Although the genetic architecture of the traits studied here was moderately complex, we found that the *MPK12* 53R allele could explain a large proportion of the genetic variation in WUE and stomatal conductance. Our finding that *MPK12 53R* underlies variation in stomatal conductance and WUE in Cape Verde is consistent with previous evidence that this specific allele is important in water balance. Further, these previous findings help contextualize our results in the natural population. Prior work provides molecular evidence that MPK12 is important for sensing and responding to drought stress by regulating the stomatal guard cell response to abscisic acid (ABA), a key phytohormone involved in abiotic stress responses (Jammes et al. 2009; Montillet et al. 2013; Salam et al. 2013). Using QTL mapping and introgression, Juenger and colleagues identified the *MPK12* locus and subsequently validated the effect of the Cvi-0 *MPK12* allele on WUE (Juenger et al. 2005; Des Marais et al. 2014). Des Marais et al. (2014) further showed that *MPK12* impacts guard cell size and behavior, and their work suggested that the CVI *MPK12* allele causes an altered response to vapor pressure deficits and abscisic acid–induced inhibition of stomatal opening. Additional analysis showed that the functional *MPK12* allele is involved in CO_2_ signaling and that the CVI *MPK12* allele has an impact that is comparable with a complete loss of function (Jakobson et al. 2016). Finally, our finding that variation in *MPK12* impacts fitness in CVI conditions is interesting in the context of previous work (Campitelli et al. 2016) demonstrating that *MPK12* variation was associated with variation in fitness components in response to a combination of drought and competition. Our study focused on the CVI natural population, which supports these previous results and connects variation in the *MPK12* gene to ecology and evolution in the natural environment.

Our results provide the potential for crop improvement in sustainable agriculture. In regions of the world where horizontal precipitation is an important source of moisture, technological approaches have been developed to collect fog for agricultural use (Klemm et al. 2012; Schemenauer, Bignell, et al. 2016; Schemenauer, Zanetta, et al. 2016). However, these are difficult to maintain and their usefulness is thus limited. A more direct approach to exploit horizontal precipitation in agricultural improvement could potentially be achieved by breeding crops with increased stomatal aperture that can better use this available resource. Future work could apply the results of studies that identify such adaptive genetic variation in local wild populations to increase crop productivity in challenging conditions. Our results suggest that breeding crops with reduced activity of MPK12 or its homologs could increase crop productivity in tropical agricultural systems, where vertical precipitation is limited and horizontal precipitation is an important component of total precipitation.

There are several open questions that could be addressed in future research. We proposed that photosynthetic efficiency should be increased in plants with increased stomatal conductance (and decreased WUE), in particular in those that carry the derived *MPK12* variant. This hypothesis could be explicitly tested in the future in a controlled study of photosynthetic efficiency. Further, although we have no specific evidence that *A. thaliana* from CVI is able to absorb water directly through the leaves, there is mounting evidence from diverse species that foliar water uptake through the leaf surface is a common strategy in humid environments where vertical precipitation is limited (Burkhardt et al. 2012; Berry et al. 2018; Binks et al. 2020). Further, there is evidence that plants in cloud forests may be especially susceptible to climate change (McDowell et al. 2008, 2011). These hypotheses could be tested in future research in controlled laboratory-based experiments as well as in field experiments in CVI.

Although the traits studied here are polygenic, our findings revealed that one variant in *MPK12* explained a substantial fraction of the trait variation. Understanding how adaptation has occurred in specific cases can inform models and predictions of how populations might generally adapt to novel environments. Although we have information about functional loci and variants from QTL mapping studies in a range of species, it has only rarely been possible to connect results from QTL studies back to the ecology of the relevant natural population. This study serves as an example of how it was possible to reconstruct the evolutionary history of a functional variant as it arose and spread across the landscape. Further, studies such as this one can inform models that aim to predict how species adapt as the environment changes or expand their ranges into more severe climates.

## Materials and Methods

### Study Populations

In this work, we used the previously released whole-genome short-read data for 189 individuals collected from 26 different locations in Santo Antão (fig. 1; supplementary Table S1, Supplementary Material online; ENA: PRJEB39079 [ERP122550]; Fulgione et al. 2022) and 61 Moroccan lines (Durvasula et al. 2017) for genetic analysis. We used the SHORE pipeline (https://github.com/HancockLab/CVI) for SNP discovery and variant calling. The variant call format (VCF) file (EVA: PRJEB44201 [ERZ1886920]; Fulgione et al. 2022) was filtered to minimize SNP calling bias and to retain only high-quality SNPs: (1) retain only bi-allelic SNPs; (2) convert heterozygous sites to missing data to mask possible false positives; (3) retain variants with coverage >3 and base quality >25. All maps were conducted using R v. 3.4.4 (R Development Core Team 2008) and the *ggmap* (Kahle and Wickham 2013) and *ggplot2* (Wickham 2016) libraries were used for plotting.

### Phenoscope Drought Experiment and Phenotyping

Trait measurement was performed using the high throughput phenotyping Phenoscope platform (https://phenoscope.versailles.inra.fr/) as previously described (Tisné et al. 2013). Santo Antão (Fulgione et al. 2022) (*n* = 152) and Moroccan (*n* = 24) *A. thaliana* lines (Brennan et al. 2014; supplementary table S1, Supplementary Material online) were grown under standard environmental conditions (8-h day/16-h night, 21 °C day/17 °C night, 65% relative humidity, and 230 µmol/m^2^ s light intensity). For each trait, two independent replicate experiments were performed. In each experiment, two replicates per genotype and two watering conditions were used. The first was a WW condition in which pots were provided with 60% of the maximum soil water content (SWC; 4.6 g H_2_O/g dry soil) not limiting for vegetative rosette growth. The second condition was a “water-deficit condition” (WD) in which pots were provided with 25% SWC (1.4 g H_2_O/g dry soil). Plants were propagated on peat moss plugs, then selected for homogeneous germination and transferred onto the Phenoscope table 8 days later, that is, 8 days after sowing (DAS). On the Phenoscope, SWC reached 60% for control-treated plants at 12 DAS and 25% for moderate-drought-treated plants at 16 DAS. At 32 DAS, the whole rosette of two replicates for each genotype per treatment was collected, ground, and analyzed for carbon isotope discrimination (δ^13^C) as an estimate of WUE. Isotope discrimination analysis was conducted at the CEPLAS Plant Metabolism and Metabolomics Laboratory, Heinrich Heine University Düsseldorf (HHU) as described previously (Gowik et al. 2011). In short, dried plant material was ground to a fine powder and analyzed using an Isoprime 100 isotope ratio mass spectrometer coupled to an elemental analyzer (ISOTOPE cube; Elementar Analysensysteme, Hanau, Germany) following the manufacturer's recommendations. The carbon isotope ratio is expressed as ‰ against the Vienna Pee Dee Belemnite standard.

We measured leaf stomatal conductance using a leaf Porometer (SC-1, Decagon Devices, Pullman, WA, USA). According to the manual guide, the Porometer device was calibrated before measurements with a 100% humidity filter paper as a reference. It was challenging to measure the rosette leaves directly due to their reduced size and the small area of the SC-1 Porometer leaf clamp. Therefore, a fully developed leaf per line and per treatment of each genotype was examined immediately after detachment. The measurements were performed across several days (29–32 DAS) around mid-day.

### Phenotype Data Analysis

Differences in the phenotype distributions were evaluated using both parametric and nonparametric tests. For conducting Wilcoxon rank sum tests, we used *wilcox.test* in the *stat_compare_means* function (“*ggpubr*” package; Kassambara 2020). We also used linear models to test fixed effects of treatment, geographic region, and their interaction on the measured phenotypic traits. For this, we used the R package *lme4* (Bates et al. 2014) to run the following model for each phenotype.


$$Y_{ijk}=\;\mu +\alpha_{i}+\;\beta_{j}+\;\gamma_{ij}+\;\varepsilon_{ijk}$$

where *Y*_*ijk*_ represents the phenotypic value; *μ* is the overall mean; *α*_*i*_ is the effect of the treatment; *β*_*j*_ is the effect of the geographic region; *γ*_*ij*_ is the interaction between treatment and region; $\varepsilon_{ijk}$ is the residuals.

The correlations between phenotypes in both treatments are Pearson correlations calculated in R using the *cor.test* function. We evaluated the significance of correlations with the *t*-test implemented in the *cor.test* function.

We obtained the individual data for the total seed number (as a proxy of fitness) from (Fulgione et al. 2022). Since no block effect was detected in the simulated CVI conditions experiment, we used the median per genotype across replicates as the phenotype. We tested the effects of the *MPK12* 53R derived variant on fitness using GLMs (R function *glm*). To correct for over-dispersion of the seed number, we used a negative binomial GLM using the “*glm.nb*()” function in the “*MASS*” v.7.3-54 package in R.

### Quantitative Genetic Analyses

We estimated heritability for traits in this study based on the proportion of the phenotypic variance explained by all genotyped SNPs, which is commonly referred to as “chip heritability” (Zhou and Stephens 2012; Zhou 2014). To perform the association analysis, we first filtered out indels and nonbiallelic SNPs from the VCF. We considered only SNPs with read coverage DP ≥ 3 and quality GQ ≥ 25. We then applied a 5% cutoff for the MAF. Subsequently, we carried out the association analyses between genomic variants and stomatal conductance and WUE as traits using the univariate LMM implemented in GEMMA (Zhou and Stephens 2012), separately for WW and WD conditions, as well as the average for each trait across both conditions and the drought response (difference between conditions: WW-WD).

According to Shim et al. (2015) and based on the GEMMA outputs, we calculated the proportion of variance in each trait explained by a given SNP (PVE) using the following equation:


$$\mathrm{PVE}=\frac{\Delta 2\;{\hat{\;\beta }}^{2}\mathrm{MAF}(1-\mathrm{MAF})}{2\;{\hat{\;\beta }}^{2}\mathrm{MAF}(1-\mathrm{MAF})+{\left(se(\;\hat{\;\beta })\right)}^{2}2N\;\mathrm{MAF}(1-\mathrm{MAF})}$$

where $\hat{\beta }$ is the effect size estimate, $se(\hat{\beta })$ is the standard error of effect size for the SNP, MAF is the MAF for the SNP, and *N* is the sample size.

To infer the genetic architecture of the traits, we used a polygenic GWA Bayesian sparse linear-mixed model (BSLMM) implemented in GEMMA (Zhou and Stephens 2012), which models the polygenic architecture as a mixture of large and small effects. BSLMM accounts for the relatedness among individuals by including a genomic kinship matrix as a random effect in the model. Furthermore, the approach accounts for the LD between SNPs by inferring locus effect sizes (β) while controlling for other variants included in the model. Using this approach, we modeled two effect hyperparameters: a basal effect (β), which captures small-effect loci that contribute to the studied trait, and an additional effect (γ), which captures a subset of loci with the most potent effects. To estimate the effects of all SNPs, the sparse effect size for each locus was calculated by multiplying (β) by (γ). We listed the variants with the highest sparse effects on the studied trait.

We then investigated the genetic correlations between traits using the multivariate model in GEMMA (Zhou and Stephens 2012; Zhou 2014). Accordingly, we conducted the correlations between the effect sizes of all loci (β) for each trait through Pearson correlations calculated in R using the *cor.test* function. We evaluated the significance of correlations with the *t*-test implemented in the *cor.test* function.

### Population Structure Analysis

In a preprocessing step before population structure analysis, we used PLINK v1.9 to prune our SNP sets for linkage disequilibrium by removing any variables with correlation coefficients (*r^2^*) >0.1 across windows of 50 Kb with a step size of 10 bp. Then, we removed variants with missing data by setting the parameter −geno to 0.

To conduct the PCA, we used the −pca option in PLINK v1.9 (Purcell et al. 2007). We produced the whole-genome neighbor-joining tree in R v3.3.4 (R Development Core Team 2008) using the packages “*APE*” v5.5 (Paradis and Schliep 2019) and “*adegenet*” v2.1.4 (Jombart 2008). To evaluate the relationships between the five Santo Antão subpopulations and visualize how the tree topology changes across the genome, we used a phylogenetic weighting approach, *Twisst* (Martin and Van Belleghem 2017). This method uses maximum likelihood topology inference across genomic windows to produce a distribution of topology weightings (Martin and Van Belleghem 2017). Starting with our LD-pruned data set, we converted our data to “.geno” format using the script “parseVCF.py” (https://github.com/simonhmartin/genomics_general/tree/master/VCF_processing), and we obtained the maximum likelihood trees in sliding windows of 50 SNPs using the script “phyml_sliding_windows.py” (https://github.com/simonhmartin/genomics_general/tree/master/phylo). Then, we ran *Twisst* on the complete set of inferred trees for the five Santo Antão subpopulations (Fi; Lombo de Figueira, Co; Cova de Paúl, Ri; Ribeira de Poio, Pi; Pico da Cruz, and Es; Espongeiro) to calculate the exact weighting of each local window. We used the Cova de Paúl subpopulation as an outgroup in this step. To plot the topologies, we used R v3.3.4 (R Development Core Team 2008) and the “*APE*” package (Paradis and Schliep 2019).

#### Inferring the Genealogical History of *MPK12* G53R

We used RELATE v1.1.4 (Speidel et al. 2019) to infer the genealogical trees for the derived *MPK12* 53R allele variant (Chr2:18947614). We used bcftools v1.9 (Li 2011) to filter the VCF file for quality, to remove nonbiallelic SNPs, to remove fixed sites, and to filter out missing data with the command: <bcftools view -m2 -M2 -v snps −min-ac = 1 -i “MIN(FMT/DP) > 3 & MIN(FMT/GQ) > 25 & F_MISSING = 0”>. Within RELATE, we used the command RelateFileFormats (using −mode ConvertFromVcf) to convert the VCF file into haplotype and sample files. We ran RELATE under a haploid model for chromosome 2 (using −mode All) and we defined parameters as follows. For the mutation rate, we corrected the estimate for *A. thaliana* of 7 × 10^−9^ derived from (Ossowski et al. 2010) for the percent missing data in 1 Mb sliding windows every 50 kb across the entire genome (2.245 × 10^−9^ for *MPK12* 53R variant). For the recombination map, we corrected a published map based on crosses (Salomé et al. 2012) for the outcrossing rate of 5% estimated in natural populations (Bomblies et al. 2010). For coalescence rates, we used the genome-wide rates inferred previously in (Fulgione et al. 2022) for the Santo Antão population. We set the generation time to 1 year. To produce genealogical trees for *MPK12* 53R variant with confidence intervals for the estimated ages based on 200 samples from the MCMC (derived using SampleBranchLengths.sh −format a, and using default settings), we used the script TreeViewSample.sh, with 10*N steps (N is the number of haplotypes) and 1,000 burn-in iterations.

### Climatic Variables

We retrieved data for the 19 bioclimatic variables (supplementary table S7, Supplementary Material online) commonly used to study the pattern of species distribution and the water vapor pressure (humidity) from the WorldClim global climate version 2 (Fick and Hijmans 2017) (https://worldclim.org/data/worldclim21.html), at a resolution of 30 s (∼1 km^2^). We also obtained site-specific data for the accumulated rainfall amount during the growing season and aridity index from CHELSA (Karger et al. 2017; supplementary Table S7, Supplementary Material online), which we used for a comparison between the climate of collection sites in Santo Antão and Moroccan sites (supplementary fig. S1, Supplementary Material online). We extracted the climatic variable values for the specific geographical coordinates for each sampling location in Santo Antão and Morocco using the “*raster*” package in R (Hijmans et al. 2015). The shift of the climate variable distribution between Santo Antão and Morocco was tested using a two-tailed Wilcoxon rank sum test with the function *wilcox.test* implemented in the *stat_compare_means* function (“*ggpubr*” package; Kassambara 2020).

### RDA: Linking Genomic Variation to Environment Predictors

We used the RDA approach implemented in the R package “*vegan*” v. 2.5-7 (Oksanen et al. 2020) to investigate the relative contributions of the bioclimatic variables and the spatial distribution of *MPK12* G53R across the Santo Antão landscape. RDA uses multiple regression to model matrices of explanatory variables (X and Y), in which X represents a set of environmental variables and Y represents a dependent matrix of genotypic data. It links genomic variation to environmental predictors while accounting for geographic population structure by including geographic distance as a model covariate. Genotype data from a set of genome-wide LD-pruned SNPs (*n* = 8,475) and environmental data (supplementary Table S7, Supplementary Material online) were analyzed by running the full model. We used analyses of variance (ANOVA with 1,000 permutations) to assess the significance of each environmental variable within the RDA model. Then we used a stepwise permutational ordination method using the ordination step “*ordistep*” function in the R package “*vegan*” v. 2.5-7 (Oksanen et al. 2020) with 1,000 permutations to evaluate the environmental parameters and identify the model that best describes the spatial distribution of the genotype data. This function selects variables to build the “optimal” model with the highest adjusted coefficient of determination (*R*_adj_^2^) and removes the nonsignificant variables one at a time using permutation tests.

### Evolutionary History of *MPK12* 53R

#### Evidence of Positive Selection

To detect signatures of positive selection in the Santo Antão population, we used three haplotype-based methods: the *EHH* (Sabeti et al. 2002), the integrated haplotype score (*iHS*; Voight et al. 2006), and cross-population EHH (*XP-EHH*; Sabeti et al. 2007) implemented in the R package “*rehh*” version 2.0.2 (Gautier et al. 2017) in R. For *iHS* and *XP-EHH*, scores were transformed for each SNP into two-sided *P*-values: p*iHS* = −log10(1–2|Φ[*iHS*]-0.5|) and *pXP-EHH* = −log10(1–2|Φ[*XP-EHH*]-0.5|), where Φ(*x*) represents the Gaussian cumulative distribution function. We used the default parameters for all analyses.

To determine whether there was enrichment of specific functional gene sets in the tail of the distribution of *iHS* scores, we conducted GO enrichment analysis. For this, we used the top 1% of SNP variants (>99% quantile based on the genome-wide empirical distribution) identified through the genome-wide *iHS* scores across the genomes of the Santo Antão population. Gene names were extracted based on the SNP position using the TAIR10 GFF3 gene annotation file through SNPEff (Cingolani et al. 2012). GO analysis was conducted using the ShinyGO web tool (http://bioinformatics.sdstate.edu/go/; Ge et al. 2020; see all results in supplementary Table S5 and fig. S12*A* and *B*, Supplementary Material online). After running the analysis, we checked that significant results were not driven by signals in clusters of genes. We did not find that any of the genes responsible for enrichments were located on the same chromosomes.

#### Inference of the Selection Coefficient

To infer a selection coefficient based on the reconstructed historical frequency trajectory for the derived *MPK12* allele (Chr2:18947614) we used CLUES (Stern et al. 2019). CLUES uses importance sampling over trees generated in RELATE to produce a posterior distribution from which a frequency trajectory can be inferred. We obtained estimates of the posterior distributions of allele frequencies over time using 200 samples from the MCMC and a recessive model. We inferred the selection coefficient jointly across two-time bins (epochs) of 1.5 kya between the present day and the time in the past when the variant arose (0–1.5 and 1.5–3 kya; supplementary Table S6, Supplementary Material online).

## Supplementary Material

msad031_Supplementary_Data

## Data Availability

All scripts for analyses and data visualization have been archived in the Github repository (https://github.com/HancockLab/CVI_WUE_MPK12_LocalAdapt).
