# Supplementary material for: Genomic Basis of Adaptation to a Novel Precipitation Regime
Source: Mol Biol Evol. 2023 Feb 15;40(3):msad031. doi: 10.1093/molbev/msad031 (PMC10037080; doi:10.1093/molbev/msad031)
Supplement: msad031_Supplementary_Data [file msad031_supplementary_data.zip › SupplementaryMaterial.pdf]

Supplementary Material for

Genomic basis of adaptation to a novel precipitation regime

Ahmed F. Elfarargi,[1] Elodie Gilbault,[2] Nina Döring,[1] Célia Neto,[1] Andrea Fulgione,[1] Andreas P. M. Weber,[3] Olivier Loudet,[2] Angela M. Hancock,[1,*]

[1]Max Planck Institute for Plant Breeding Research, 50829 Cologne, Germany.

[2]Université Paris-Saclay, INRAE, AgroParisTech, Institut Jean-Pierre Bourgin (IJPB), 78000 Versailles, France

[3]Institute of Plant Biochemistry, Cluster of Excellence on Plant Science (CEPLAS), Heinrich Heine University, 40225 Düsseldorf, Germany

[*]Correspondence to: hancock@mpipz.mpg.de

This PDF file includes:

Figures S1 to S16

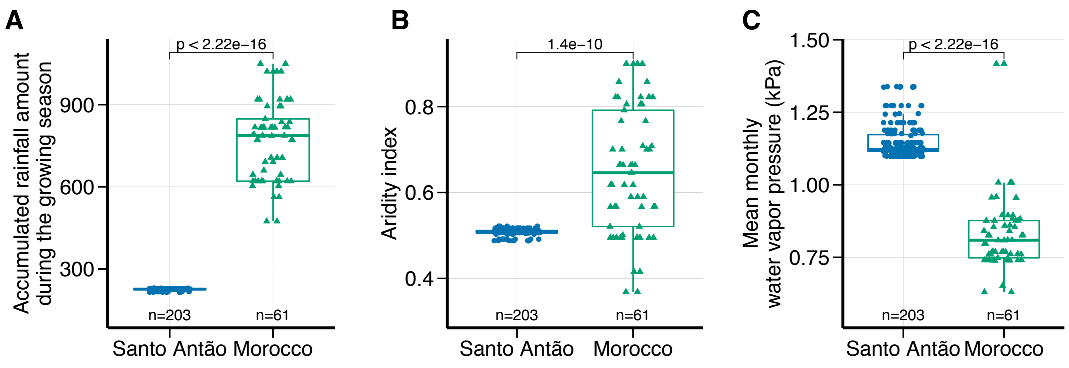

Supplementary Figure S1. *Climate of (A) accumulated rainfall during growing season (kg m^{-2} gsl^{-1}), (B) aridity index, and (C) mean monthly water vapor pressure (KPa) at collection sites in Santo Antão (blue; n=203) relative to Moroccan sites (green; n=61). The line in the center of the boxplots represents the median, the box edges represent the 25th and 75th percentiles (lower and upper bound, respectively), and the whiskers represent 95% CI. The P-values for the Mann-Whitney-Wilcoxon test are shown.*

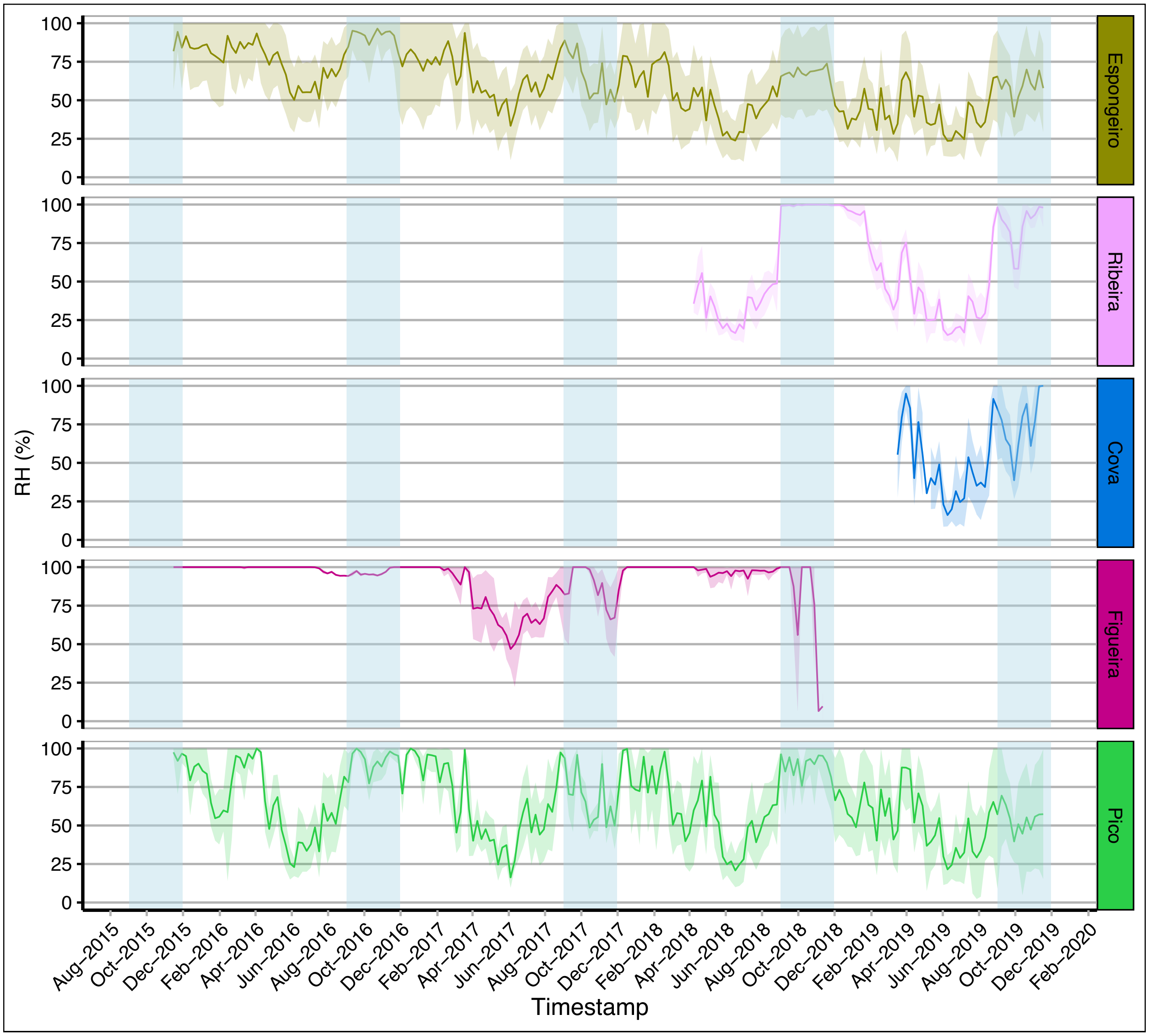

Supplementary Figure S2. *Measurements of the relative humidity (RH) over time using loggers in the field sites of the five Santo Antão sub-populations. The light blue shaded areas over time represent the growth season period (September to November).*

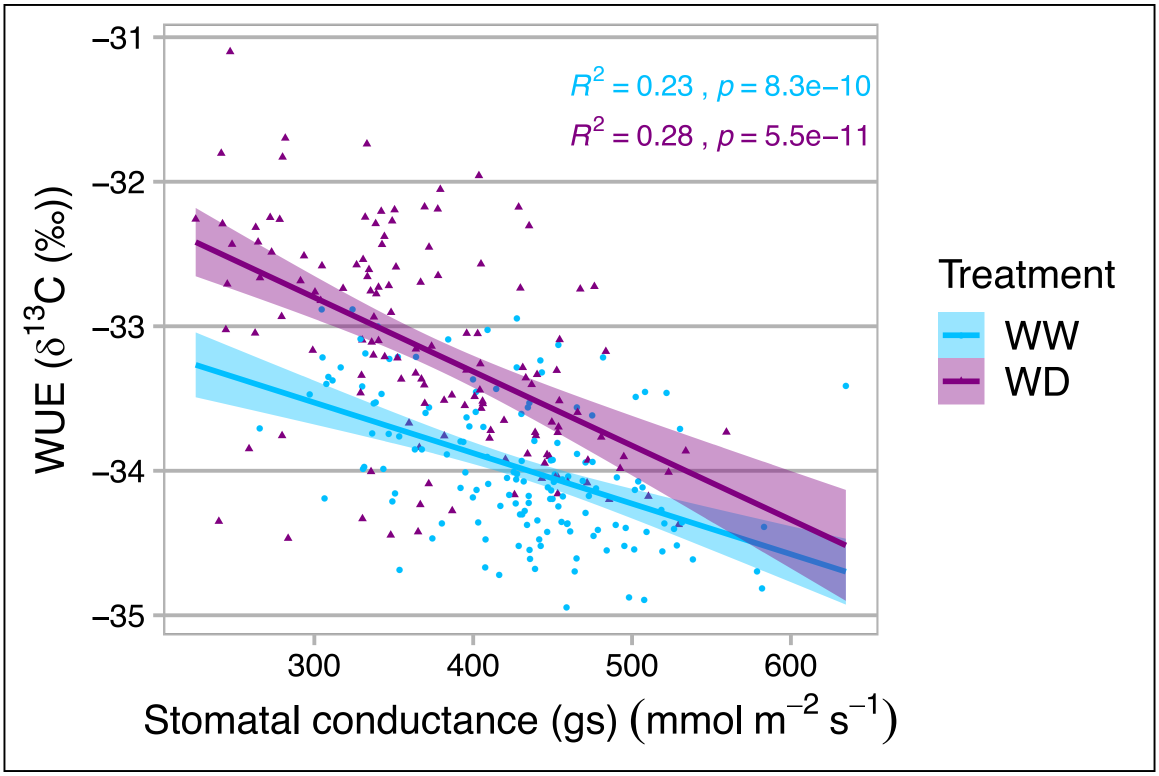

Supplementary Figure S3. *The relationship between water use efficiency (WUE) and stomatal conductance in Santo Antão population. WUE is negatively correlated with stomatal conductance in well-watered (WW) and in response to water deficit (WD). The WUE is measured as carbon isotope discrimination ($\delta^{13}C$), and the carbon isotope ratio is expressed as (per mil, ‰). The shaded areas around the slope represent the confidence interval of the correlation coefficient at 95%. R^2= Pearson's squared correlation coefficient and p= p-value.*

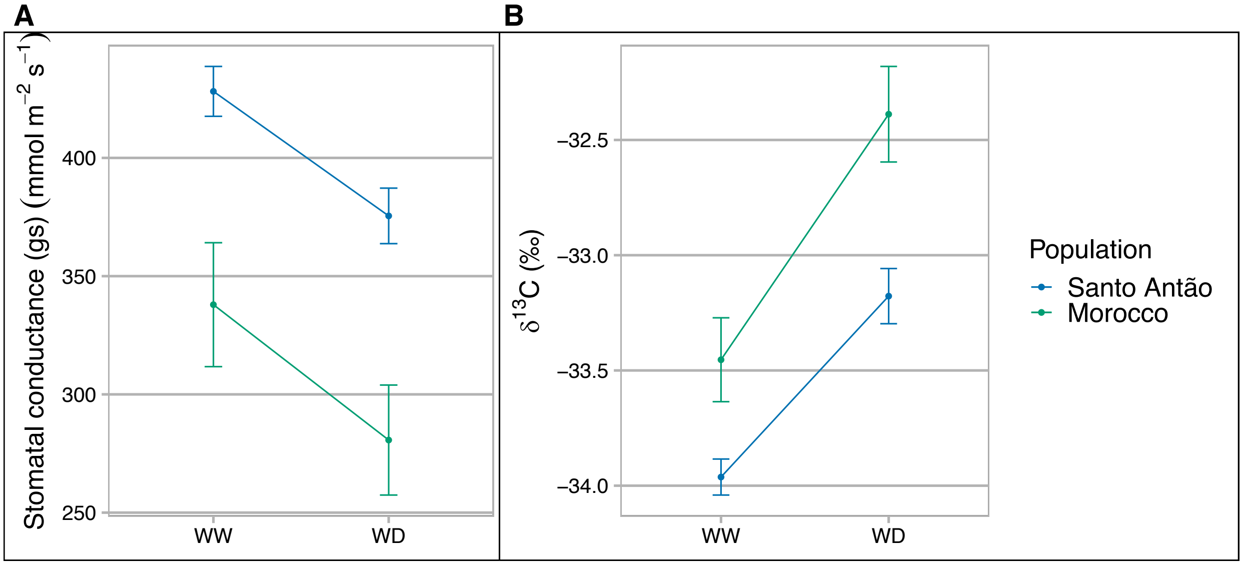

Supplementary Figure S4. *Phenotypic plasticity of (A) water use efficiency (WUE) and (B) stomatal conductance, among Santo Antão A. thaliana population under well-watered (WW) and water deficit (WD) conditions. The WUE is measured as carbon isotope discrimination ($\delta^{13}C$), and the carbon isotope ratio is expressed as (per mil, ‰). The points represent the means and the whiskers represent the 95% CI.*

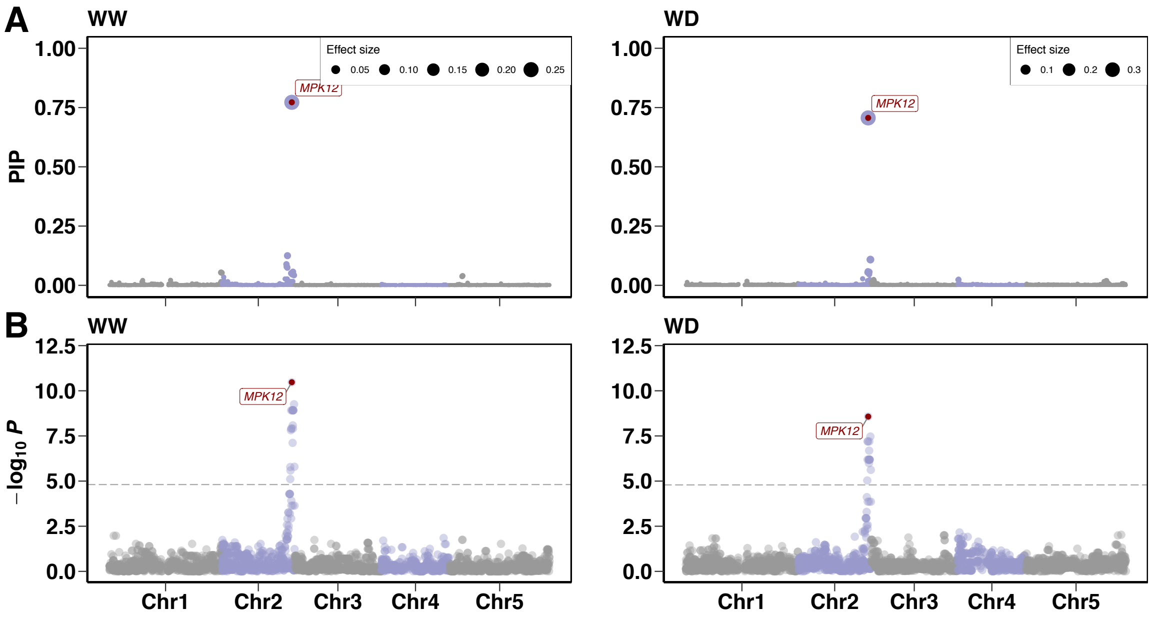

Supplementary Figure S5. Genome-wide association (GWA) mapping. *(A) Polygenic modeling of water use efficiency (WUE) variation in the Santo Antão A. thaliana population in well-watered (WW) and water deficit (WD) conditions using BSLMM. The y-axis represents the posterior inclusion probability (PIP) and the size of symbol denotes the effect size. (B) Genome-wide association mapping of WUE variation in WW and WD conditions using LMM. The horizontal dashed line corresponds to the Bonferroni genome-wide significance. In* ***A*** *and* ***B****, points represent SNPs along the five chromosomes. The red point represents the MPK12 G53R variant (a substitution of arginine for glycine at amino acid position 53).*

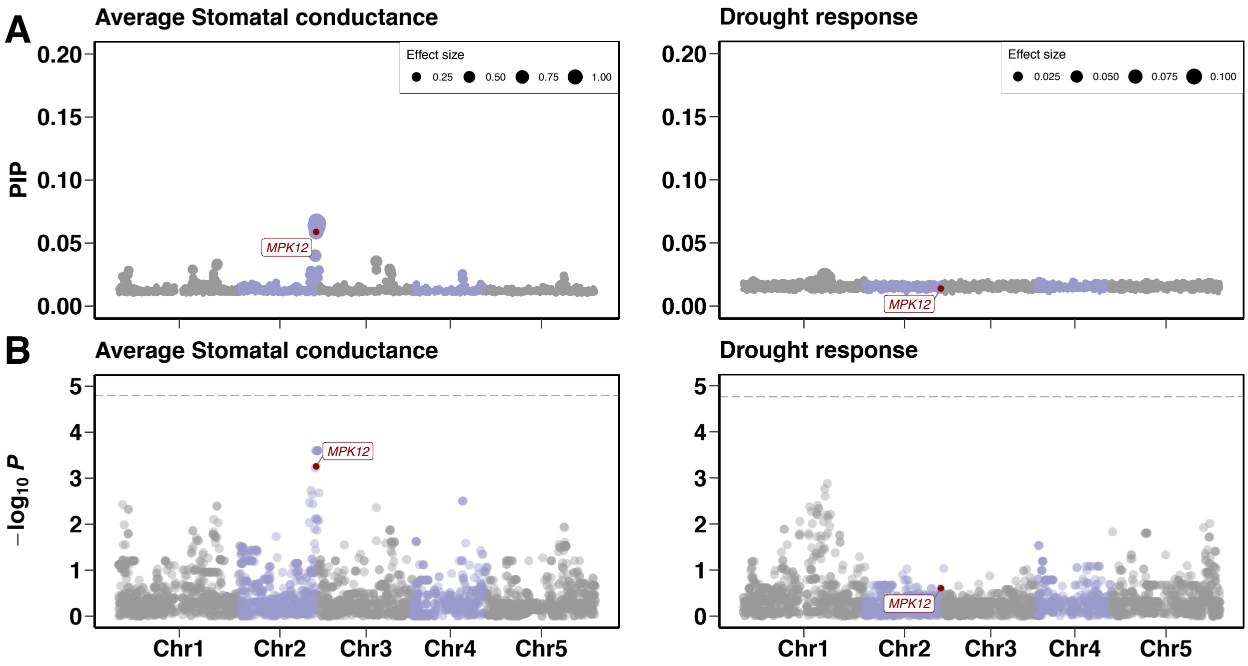

Supplementary Figure S6. Genome-wide association (GWA) mapping of stomatal conductance. *(A) Polygenic modeling of the average stomatal conductance across the well-watered (WW) and water deficit (WD) conditions and the drought response of stomatal conductance (the difference between both conditions: WW-WD) in the Santo Antão A. thaliana population using BSLMM. The y-axis represents the posterior inclusion probability (PIP) and the size of symbol denotes the effect size. (B) Genome-wide association mapping of average stomatal conductance and its drought response using LMM. The horizontal dashed line corresponds to the Bonferroni significance threshold at α = 0.05. In A and B, points represent SNPs along the five chromosomes. The red point represents the MPK12 G53R variant (a substitution of arginine for glycine at amino acid position 53).*

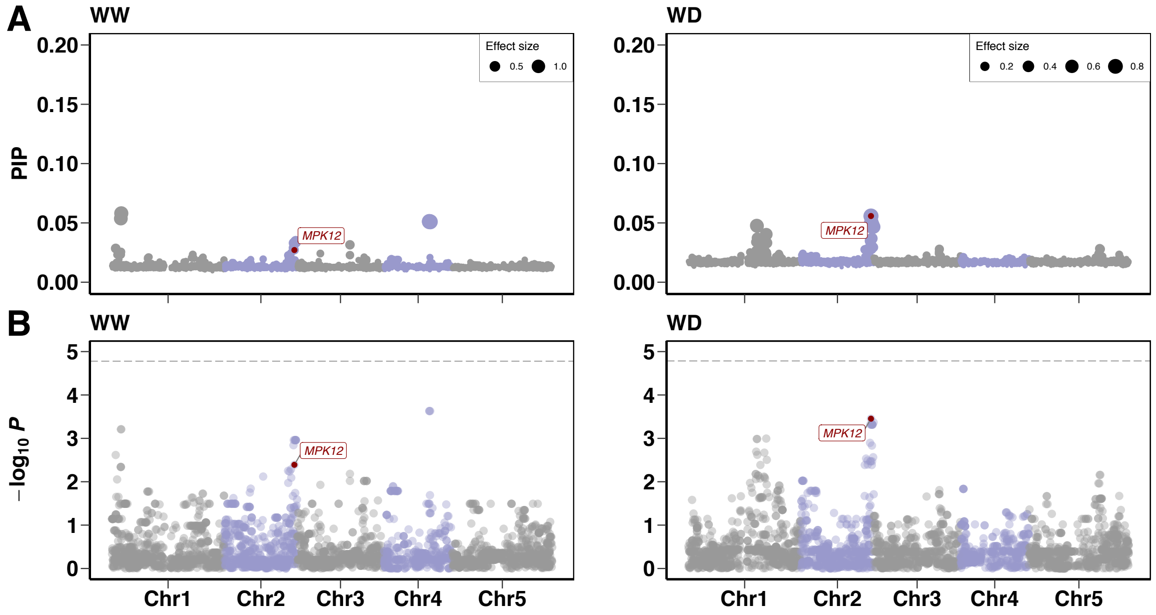

Supplementary Figure S7. *(A) Polygenic modeling of stomatal conductance variation in the Santo Antão A. thaliana population in well-watered (WW) and water deficit (WD) conditions using BSLMM. The y-axis represents the posterior inclusion probability (PIP) and the size of symbol denotes the effect size. (B) Genome-wide association mapping of stomatal conductance variation in WW and WD conditions using LMM. The horizontal dashed line corresponds to the Bonferroni significance threshold at α = 0.05. In A and B, points represent SNPs along the five chromosomes. The red point represents the MPK12 G53R variant (a substitution of arginine for glycine at amino acid position 53).*

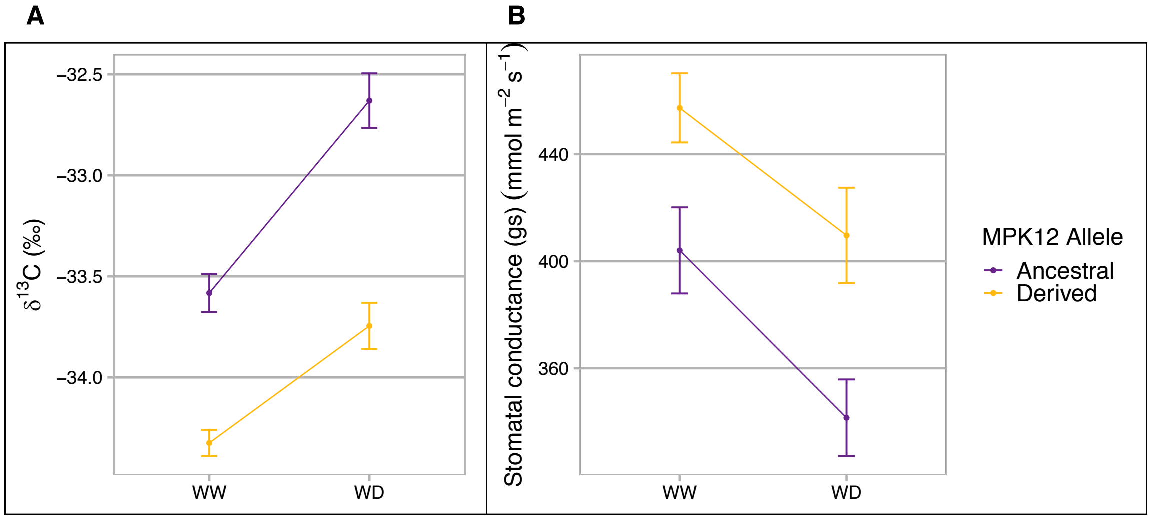

Supplementary Figure S8. *The allelic effects of MPK12 G53R on phenotypic plasticity, (A) water use efficiency (WUE) and (B) stomatal conductance, among Santo Antão A. thaliana population under well-watered (WW) and water deficit (WD) conditions. The WUE is measured as carbon isotope discrimination ($\delta^{13}C$), and the carbon isotope ratio is expressed as (per mil, ‰). The points represent the means and the whiskers represent the 95% CI.*

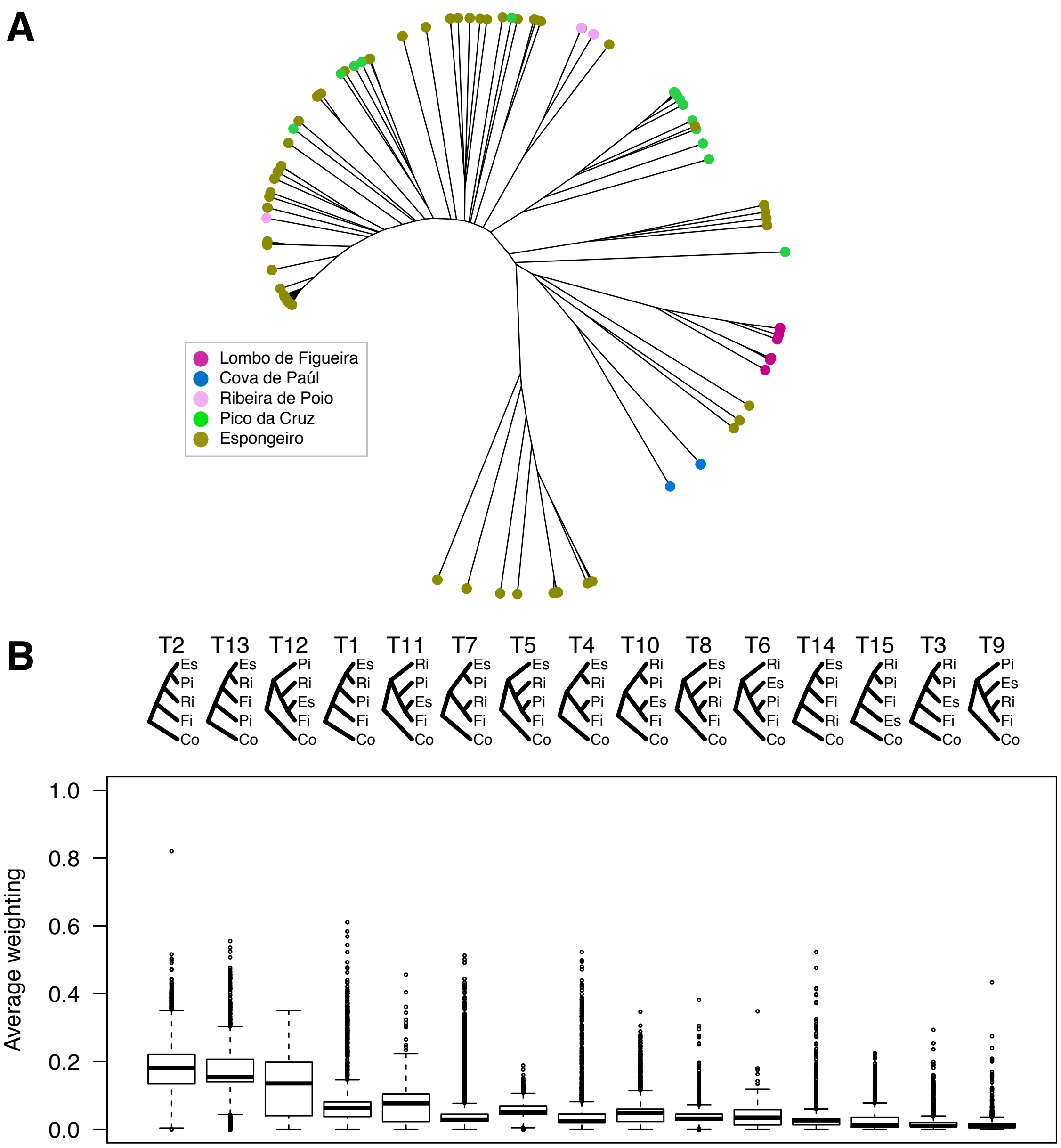

Supplementary Figure S9. History of the Santo Antão A. thaliana population. *(A) Neighbor-joining tree of Santo Antão population (n=189). Colors represent five sub-populations: Lombo de Figueira (in magenta), Cova de Paúl (in blue), Ribeira de Poio (in amethyst), Pico da Cruz (in green), and Espongeiro (in mustard). (B) Genome-wide Twisst analysis showing the average topology weighting (y-axis) among the fifteen possible phylogenetic topologies using sliding windows containing 50 variable sites. The trees are ordered based on their average topology weightings with the highest topology weighting for topology 2 (T2).*

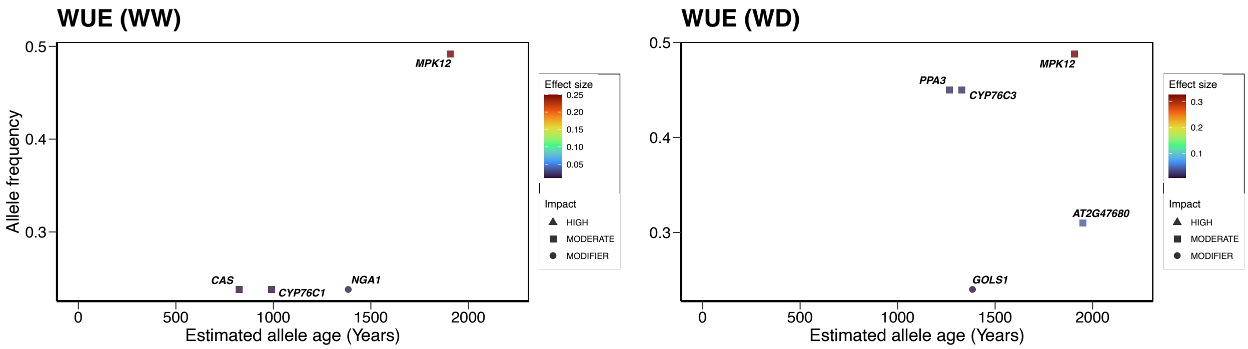

Supplementary Figure S10. *Relationship between estimated allele ages (inferred in RELATE) and allele frequencies of variants with major effects estimated with polygenic GWA mapping for water use efficiency (WUE) in well-watered (WW) and water deficit (WD) conditions in the Santo Antão population. Color denotes variant effect size from the BSLMM and shape denotes predicted impact from gene annotation.*

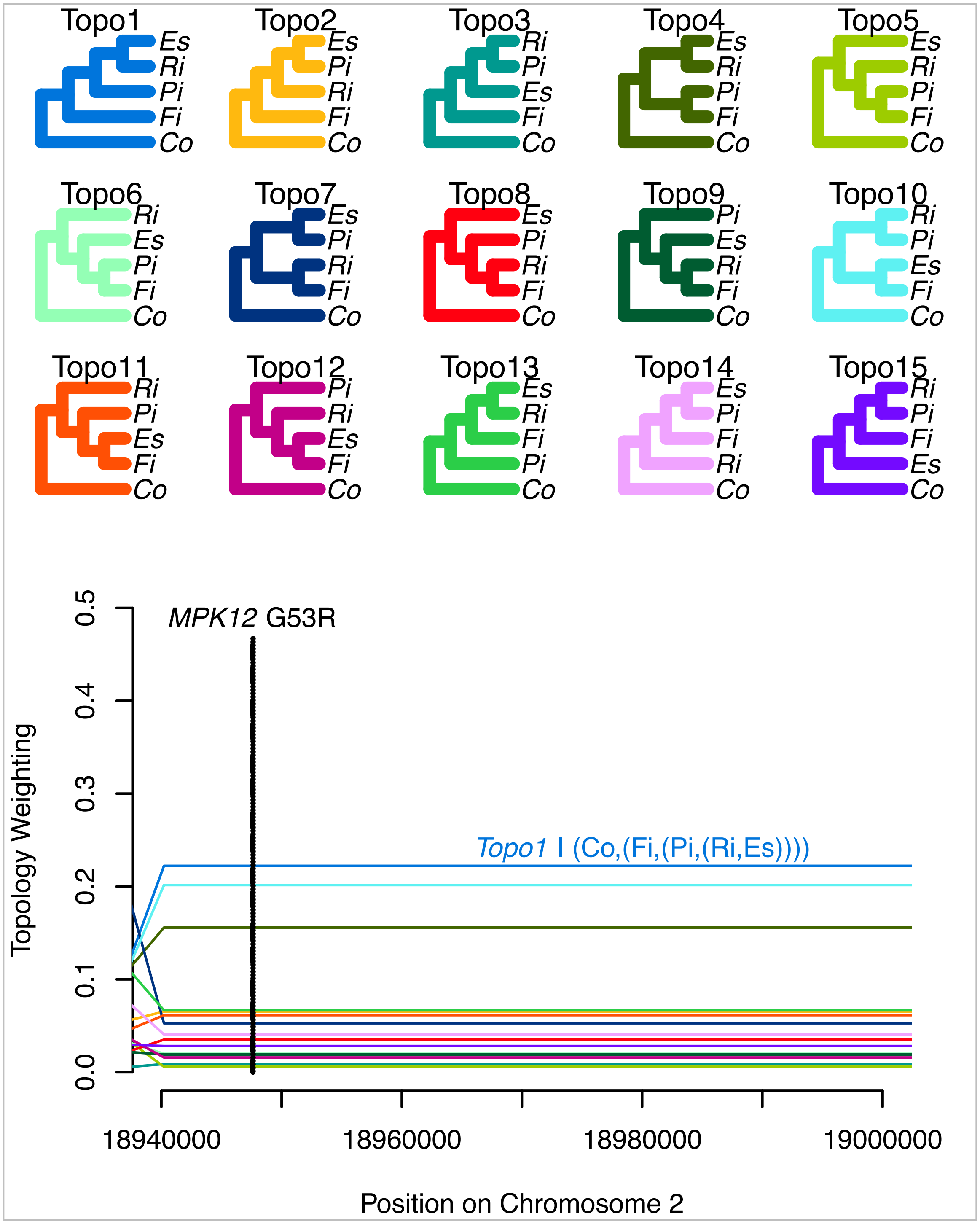

Supplementary Figure S11. *Genome-wide phylogenetic analysis inferred by Twisst, focusing on the MPK12 genomic region. Topology 1 (Topo1) has the highest topology weighting (Bottom panel) for the region surrounding MPK12 G53R among the fifteen possible phylogenetic topologies (Top panel; as previously shown in Supplementary Figure. S9B).*

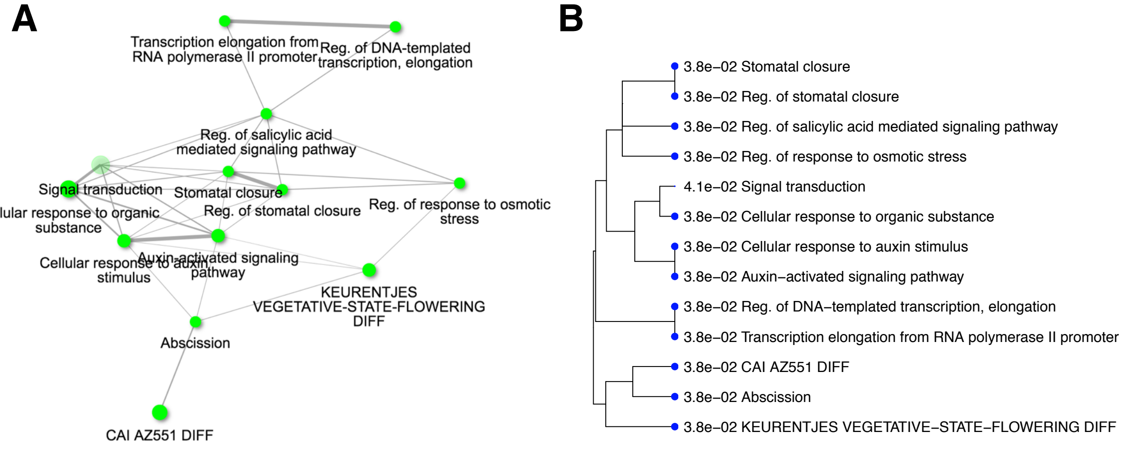

Supplementary Figure S12. *Gene ontology (GO) analysis of the top 1% of genes identified through the genome-wide integrated haplotype score (iHS) across the genomes of the Santo Antão population. (**A**) Significant enriched GO molecular component terms were visualized as a network, in which related GO terms are connected by a line, whose thickness reflects percent of overlapping genes. The size of the node corresponds to number of genes. (B) A hierarchical clustering tree shows the relatedness between GO terms, in which related GO terms are grouped together based on how many genes they share. The size of the solid circle corresponds to the enrichment FDR (see results in supplementary table S5).*

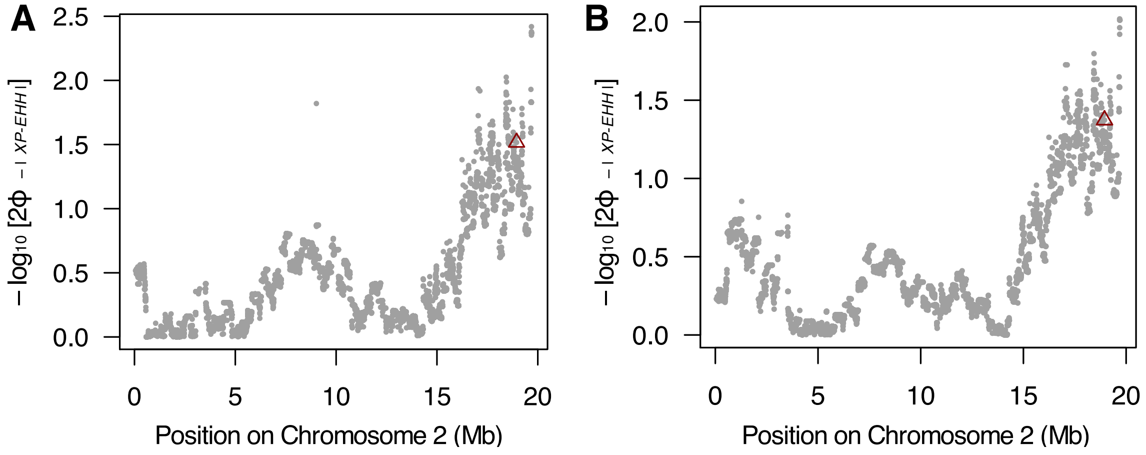

Supplementary Figure S13. *The cross-population extended haplotype homozygosity (XP-EHH) analyses for between Cova de Paúl and Espongeiro (A) and between Pico da Cruz and Espongeiro (B). A dark red triangle marks the derived MPK12 53R variant.*

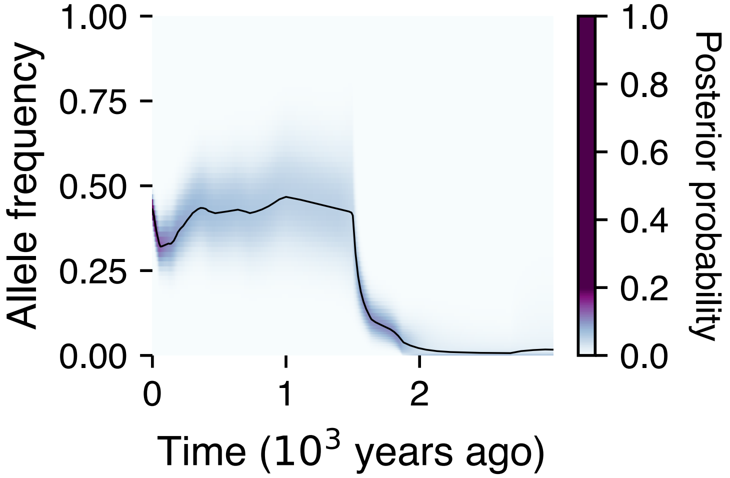

Supplementary Figure S14. *Inferred allele frequency trajectory for the derived MPK12 allele variant in Santo Antão. The black line corresponds to the inferred allele frequency change over time and the surrounding color to the posterior probability.*

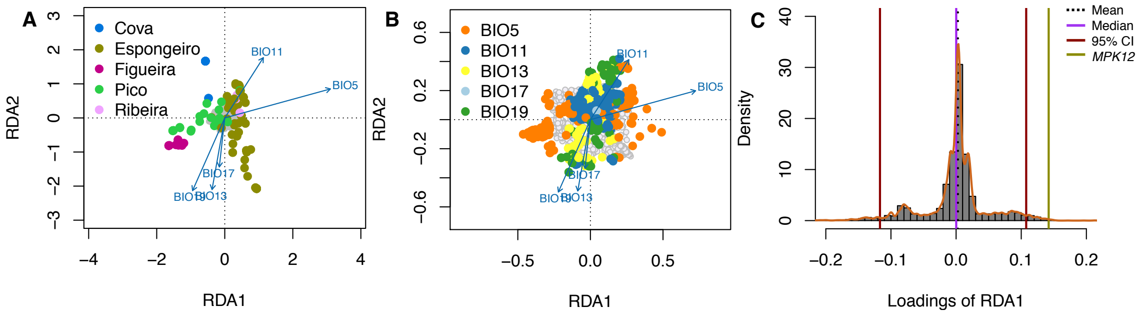

Supplementary Figure S15. Redundancy Analysis (RDA) for Santo Antão population. *(A) A biplot summarizing the result of full RDA on the bioclimatic environmental variables among the five sub-populations in Santo Antão. Individual scores for RDA axes are represented by dots, colored as the same coloring scheme for the previous figures (Lombo de Figueira: magenta, Cova de Paúl: blue, Ribeira de Poio: amethyst, Pico da Cruz: green, and Espongeiro: mustard). Grey dots represent SNPs. (B) A biplot summarizing the result of full RDA among the five bioclimatic variables for the outlier SNPs detected with the RDA outlier detection approach. The coloring scheme is for the SNPs based on the bioclimatic variable that is most strongly correlated with, and grey dots represent the non-candidate SNPs. In A and B, the light blue arrows represent the bioclimatic variables, where the arrow length reflects the amount of variance in SNP genotypes explained by that bioclimatic variable, and the angles represent the correlation between bioclimatic variables. The illustrated axes were statistically significant (P < 0.05). (C) Histogram of the SNP loadings on RDA1. Dashed black, purple, and red lines represent the mean, the median, and 95% of confidence intervals (CI), respectively. The yellow line represents the loading of the MPK12 derived allele variant on the tail of RDA1. Abbreviations:* ***BIO5****, Max Temperature of Warmest Month;* ***BIO11****, Mean Temperature of Coldest Quarter;* ***BIO13****, Precipitation of Wettest Month;* ***BIO17****, Precipitation of Driest Quarter;* ***BIO19****, Precipitation of Coldest Quarter.*

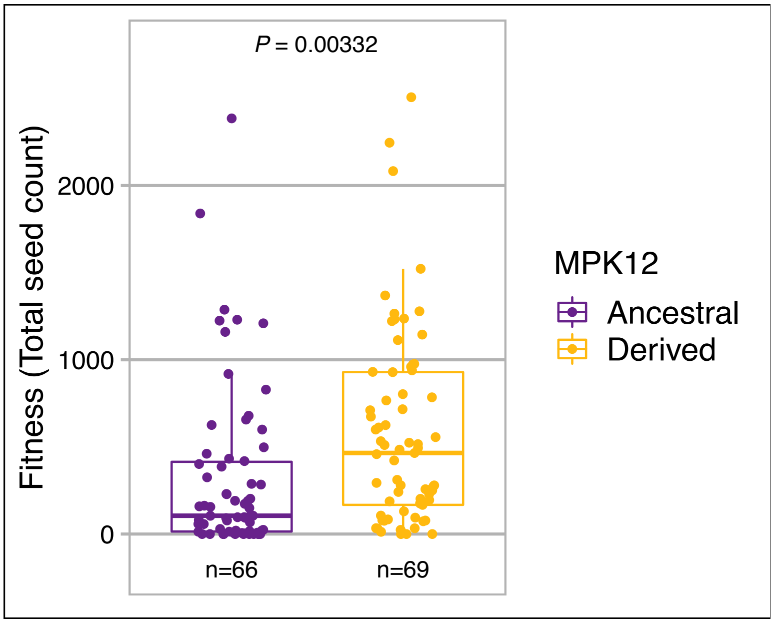

Supplementary Figure S16. *Phenotypic effects of MPK12 variants on fitness (total seed count) among Santo Antão A. thaliana population under simulated Cape Verde growth conditions. The P-value represents the fitness effect of the derived MPK12 allele, using a negative binomial GLM model (supplementary table S11). The line in the center of the boxplots represents the median, the box edges represent the 25th and 75th percentiles (lower and upper bound, respectively), and the whiskers represent 95% CI. Each dot represents the median across four replicates per accession.*
